# Knockdown of UTX/KDM6A Enriches Precursor Cell Populations in Urothelial Cell Cultures and Cell Lines

**DOI:** 10.3390/cancers12041023

**Published:** 2020-04-21

**Authors:** Alexander Lang, Patcharawalai Whongsiri, Merve Yilmaz, Tobias Lautwein, Patrick Petzsch, Annemarie Greife, Cagatay Günes, Karl Köhrer, Günter Niegisch, Michèle Hoffmann, Wolfgang A. Schulz

**Affiliations:** 1 Department of Urology, Medical Faculty, Heinrich Heine University Düsseldorf, 40225 Düsseldorf, Germany; alexander.lang@hhu.de (A.L.); pawho100@hhu.de (P.W.); meyil108@hhu.de (M.Y.); guenter.niegisch@med.uni-duesseldorf.de (G.N.); michele.hoffmann@hhu.de (M.H.); 2 Department of Cardiology, Pulmonology, and Vascular Medicine, Medical Faculty, Heinrich Heine University Düsseldorf, 40225 Düsseldorf, Germany; 3 Genomics Transcriptomics Lab, Biomedical Research Center, Heinrich Heine University Düsseldorf, 40225 Düsseldorf, Germany; tobias.lautwein@hhu.de (T.L.); patrick.petzsch@hhu.de (P.P.); koehrer@hhu.de (K.K.); 4 Chair for Molecular Physical Chemistry, Heinrich Heine University Düsseldorf, 40225 Düsseldorf, Germany; annemarie.greife@hhu.de; 5 Department of Urology, Medical Faculty, Ulm University, 89069 Ulm, Germany; cagatay.guenes@uniklinik-ulm.de

**Keywords:** urothelium, urothelial stem cells, bladder cancer, histone modification, histone demethylase, chromatin regulators, urothelial carcinoma, p53, cytokeratin 14

## Abstract

The histone demethylase UTX (gene: *KDM6A*) directs cell and tissue differentiation during development. Deleterious mutations in *KDM6A* occur in many human cancers, most frequently in urothelial carcinoma. The consequences of these mutations are poorly understood; plausibly, they may disturb urothelial differentiation. We therefore investigated the effects of UTX siRNA-mediated knockdown in two in vitro models of urothelial differentiation; namely, primary cultures of urothelial epithelial cells treated with troglitazone and PD153035 and the immortalized urothelial cell line HBLAK treated with high calcium and serum. In both models, efficient UTX knockdown did not block morphological and biochemical differentiation. An apparent delay was due to a cytotoxic effect on the cell cultures before the initiation of differentiation, which induced apoptosis partly in a p53-dependent manner. As a consequence, slowly cycling, smaller, KRT14^high^ precursor cells in the HBLAK cell line were enriched at the expense of more differentiated, larger, proliferating KRT14^low^ cells. UTX knockdown induced apoptosis and enriched KRT14^high^ cells in the BFTC-905 papillary urothelial carcinoma cell line as well. Our findings suggest an explanation for the frequent occurrence of *KDM6A* mutations across all stages and molecular subtypes of urothelial carcinoma, whereby loss of UTX function does not primarily impede later stages of urothelial differentiation, but favors the expansion of precursor populations to provide a reservoir of potential tumor-initiating cells.

## 1. Introduction

UTX (ubiquitously transcribed tetratricopeptide repeat, X chromosome) is encoded by the gene *KDM6A,* located on the X chromosome. *KDM6A* is frequently affected by deleterious mutations in urothelial carcinoma (UC) and other cancers. UTX is therefore considered a tumor suppressor [1]. Its mode of action is not fully understood and may differ between cancer types [2,3]. UTX has several molecular functions, including, prominently, a specific histone demethylase activity towards dimethylated or trimethylated lysine 27 of histone H3 (H3K27me2/3) [4,5]. UTX participates in the MLL2/3 complex (also known as COMPASS-like), which catalyzes H3K4 methylation, and in interactions with the chromatin remodeling SWI/SNF complex and the histone acetyltransferase CBP [1]. During fetal development, UTX modulates stem cell differentiation and HOX gene regulation [5,6]. It is therefore plausible to assume that UTX inactivation in urothelial carcinoma might promote cancer development via aberrant urothelial differentiation. This idea is supported by observations in other cancer types. For instance, loss of UTX in myeloid leukemia leads to dysregulation of transcription factor programs steering the differentiation of hematopoietic cells [7,8]. Similarly, in the pancreas, UTX deficiency results in squamous metaplasia and cancer by deregulation of tissue-specific enhancer activities [9]. However, *KDM6A* mutations are found across all molecular subtypes of invasive UC [10] and are even frequent in well-differentiated papillary UC [11], as reviewed in [2]. To date, there is no direct evidence on whether and to which extent urothelial differentiation is disturbed by UTX loss of function. 

To address this question, we used two models of urothelial differentiation. First, primary cultures of normal urothelial cells (UECs) derived from ureters of nephrectomy patients consist mainly of cells with a basal phenotype (KRT14-/KRT5+/KRT20-) and a variable proportion of KRT14+/KRT5+/KRT20- cells, which are regarded as stem cells in the urothelium [12,13,14,15,16,17]. Treatment with a PPARγ agonist (troglitazone) and the EGF receptor inhibitor PD153035 (TZ/PD protocol) induces biochemical markers of urothelial differentiation, such as KRT20 and uroplakins, e.g., UPK2, while decreasing KRT14 and KRT5 expression [18]. Alternatively, urothelial differentiation can be elicited by increasing the Ca^2+^ concentration in the culture medium and adding calf serum (Ca/FCS protocol) [19]. The spontaneous immortalized urothelial cell line HBLAK provides a more conveniently available model than primary urothelial cultures, but in these cells the Ca/FCS protocol is more efficacious than the TZ/PD protocol [20]. Like UEC cultures, HBLAK contains a subpopulation of KRT14+/KRT5+/KRT20− cells (hereafter KRT14^high^ cells), and upon Ca/FCS treatment yields a high percentage of cells expressing KRT20 and UPK2, whereas KRT14^high^ cells decrease in proportion. 

Here, we studied the effect of efficient UTX siRNA-mediated knockdown on TZ/PD-induced differentiation of UECs and on Ca/FCS-induced differentiation of HBLAK cells. Unexpectedly, we did not observe a major effect on differentiation in either cell model, but increased apoptotic cell death prior to and independent of differentiation induction, which was partly mediated by p53 activation. Interestingly, cell death resulted in an increased ratio of KRT14^high^ over KRT14^low^ cells. Therefore, we characterized these two populations in more detail in the HBLAK cell line. Finally, we observed an analogous effect of UTX knockdown in the BFTC-905 urothelial carcinoma cell line, which also contains KRT14^high^ and KRT14^low^ cells.

## 2. Results

### 2.1. Efficiency of UTX Knockdown

UTX was detectable in HBLAK cells and in many urothelial carcinoma cell lines as an approximately 138 kDa band by western blotting, at in general comparable levels (Figure S1a). In the T-24 cell line with a homozygous truncating *KDM6A* mutation, a weak band at approximately 100 kDa may correspond to the expected truncated protein. Following CRISPR/Cas-mediated *KDM6A* knockout in the SW1710 cell line (as described in [21]) UTX protein became undetectable (Figure S1b). Treatment of HBLAK cells with siRNA directed against *KDM6A*/UTX mRNA (siRNA 01) substantially decreased mRNA levels and almost completely obliterated protein expression after 2 d (Figure S1c,d). Accordingly, H3K27me3 levels increased (Figure S1e).

### 2.2. UTX Knockdown Does Not Block Urothelial Differentiation

UEC cultures consist mainly of basal urothelial cells, marked by KRT5, of which a variable fraction in each individual culture acquire the KRT14 stem cell marker [22]. HBLAK cells stain uniformly positive for KRT5 and a fraction of cells additionally express KRT14 (KRT14^high^ cells) [20]. In both cell lines, following induction of differentiation, KRT14 expression decreases, whereas KRT20 and UPK2 become significantly expressed. 

**Figure 1:**
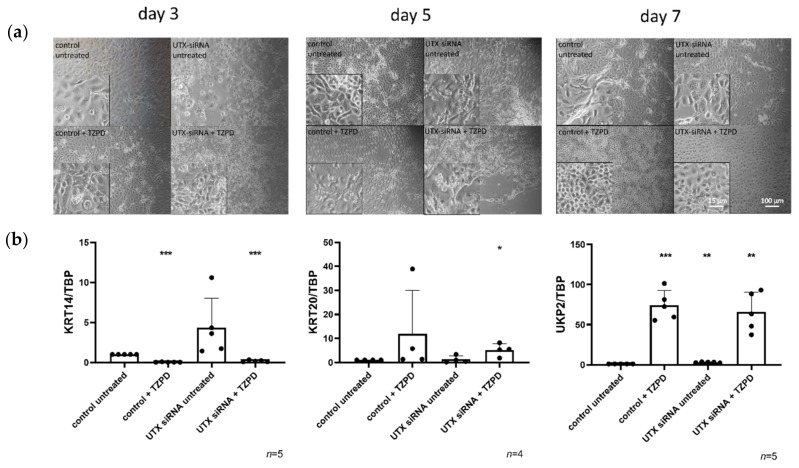
UTX knockdown in primary urothelial cells (UECs) causes delays, but does not block induction of differentiation by TZ/PD. (**a**) Morphology of UECs treated with control siRNA or UTX-siRNA 01 after 3, 5 and 7 days. Note changes in cell morphology and viability following treatment with UTX-siRNA in cells not treated with TZ/PD and a slight retardation of morphological differentiation over the first five days compared to cells treated with the control siRNA. (**b**) Expression of KRT14, KRT20 and UPK2 on day 10 after induction of differentiation. Note the lack of significant differences in the three markers in differentiated cells, but the upregulation of KRT14 mRNA following UTX knockdown in cells not treated with TZ/PD. Number of analyzed independent cultures, *n* = 4 or 5. Statistics were performed with a one sample *t* test against the set value of 1 for the untreated control (* *p* < 0.05; ** *p* < 0.01; *** *p* < 0.001). Scale 15 µm or 100 µm.

**Figure 2:**
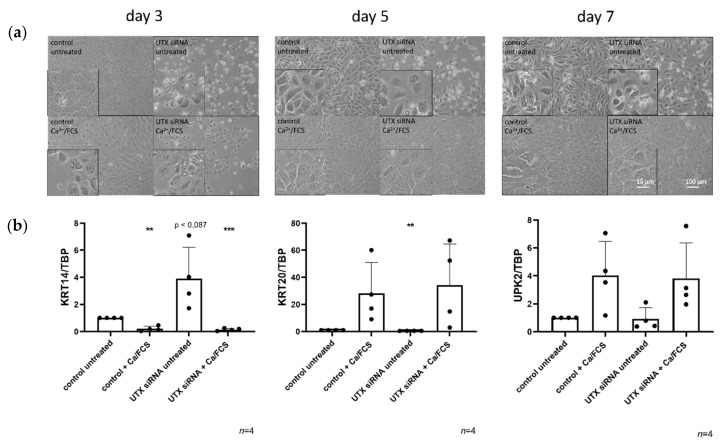
UTX-knockdown in HBLAK cells does not block differentiation induced by Ca/FCS but reduces viability. *(***a***)* Morphology of HBLAK cells treated with control siRNA or UTX-siRNA 01 after 3, 5 and 7 days. A slight retardation of morphological differentiation induced by Ca/FCS can be observed in UTX-siRNA treated cells compared to the control in the first five days. Note the changes in number and viability in cells not treated with Ca/FCS following UTX knockdown. *(***b***)* Expression of KRT14, KRT20 and UPK2 on day 10 after induction of differentiation. Note the lack of significant differences in the three markers in differentiated cells, but the relative upregulation of KRT14 mRNA following UTX knockdown in cells not treated with Ca/FCS. Number of analyzed independent experiments, *n* = 4 or 5. Statistics were performed with a one sample t test against the set value of 1 for the untreated controls (** *p* < 0.01; *** *p* < 0.001). Scale 15 µm or 100 µm.

Two days after transfection with control siRNA or UTX-siRNA, primary urothelial cells and HBLAK cells were treated with TZ/PD (UEC) or Ca/FCS (HBLAK) to induce differentiation. In both cell models, morphological examination revealed that at this time the dense monolayer was disrupted, and the number of cells was decreased after UTX knockdown, with morphological signs of apoptosis (Figure 1 and Figure 2). After induction of differentiation, the morphology of cells treated with control siRNA changed as expected over time, whereas morphological differentiation appeared delayed after previous treatment with UTX siRNA, which may be explained by the lower density of the cultures (Figure 1a and Figure 2a). Regardless, at the final time point of differentiation, no significant difference in cell morphology or expression of the differentiation marker genes *KRT14*, *KRT20* or *UPK2* could be observed between cells pretreated with control siRNA or UTX-siRNA (Figure 1b and Figure 2b). Of note, UTX mRNA expression remained low for several days into the period induction of differentiation (Figure S1c). Thus, as expected, KRT14 mRNA decreased, while KRT20 and UPK2 mRNAs increased following induced differentiation after UTX knockdown. Unexpectedly, however, an increase of KRT14 mRNA was detected in both undifferentiated UEC and HBLAK cells after UTX knockdown (Figure 1b and Figure 2b).

### 2.3. UTX Knockdown Reduces Viability in Primary Urothelial and HBLAK Cells

**Figure 3:**
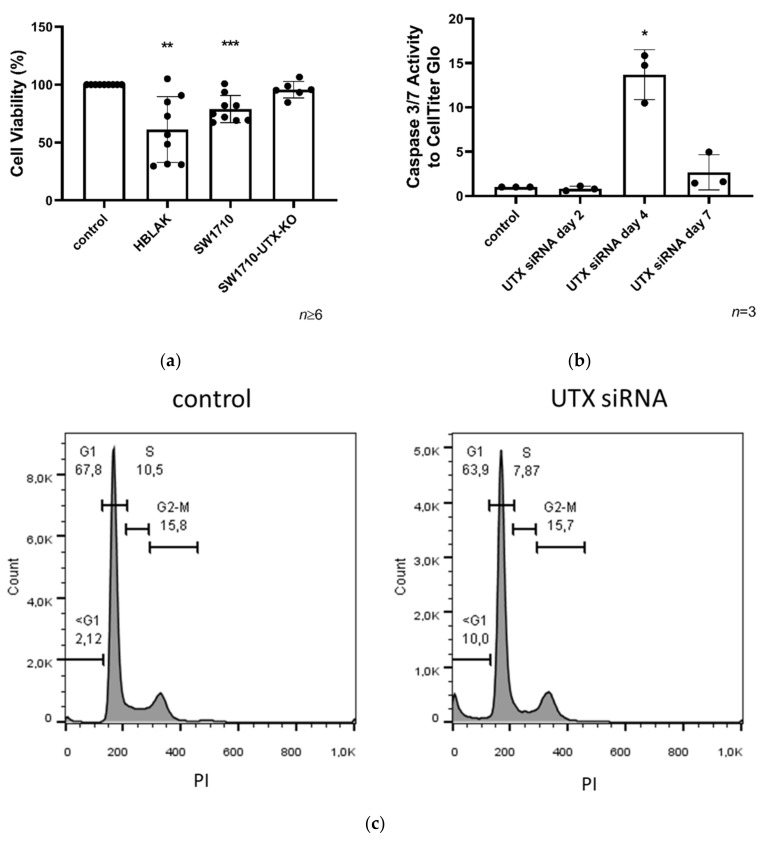
Cell viability in HBLAK and SW1710 UC cells 3 days after UTX knockdown. (**a**) Cell viability was measured via MTT on day 3 after UTX knockdown using siRNA 01 in HBLAK, SW1710 and SW1710-UTX-KO cells (*n* = 6–9). (**b**) Time course of caspase-Glo 3/7 activity relative to total viable cells measured by CellTiter-Glo assay following UTX knockdown in HBLAK cells (*n* = 3). (**c**) Cell cycle profile in HBLAK 2 d after UTX knockdown showing an increased sub-G1 fraction and a reduced G1 fraction. Note the lack of change in the G2/M fraction. Statistics were performed with a one sample *t* test against the set value of 100 against the untreated control (* *p* < 0.05; ** *p* < 0.01; *** *p* < 0.001).

The negative effect of UTX-knockdown on cell viability observed by microscopy was confirmed by MTT assays. A significant reduction of cellular viability was observed in HBLAK cells treated with UTX-siRNA for three days (Figure 3a). This finding was validated with a second, different siRNA directed against UTX (siRNA 20) in HBLAK cells (Figure S2). To further prove the specificity of the siRNA effect, the SW1710 cell line, which is wild-type for UTX, and its CRISPR/Cas-induced UTX-knockout variant (Figure S1b) were used. UTX knockdown had a slight, but significant effect in the UTX wild-type cell line, which was obliterated in the knockout cell line (Figure 3a). An assay for caspase-3/7 activity suggested that decreased cell viability in HBLAK cells treated with UTX-siRNA was at least partly due to apoptosis, which peaked on day 4 following UTX knockdown (Figure 3b). Accordingly, cell cycle analysis by flow cytometry revealed an increased sub-G1 fraction in UTX knockdown cells at day 2 (Figure 3c).

### 2.4. UTX Knockdown Increases the Fraction of KRT14^high^ Cells

**Figure 4:**
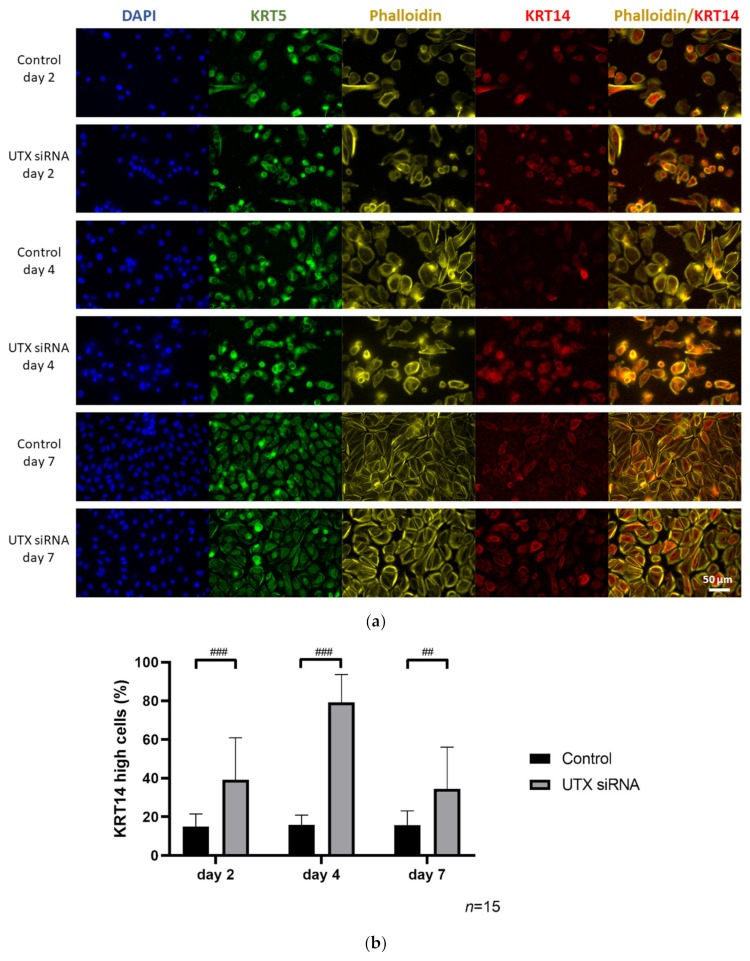

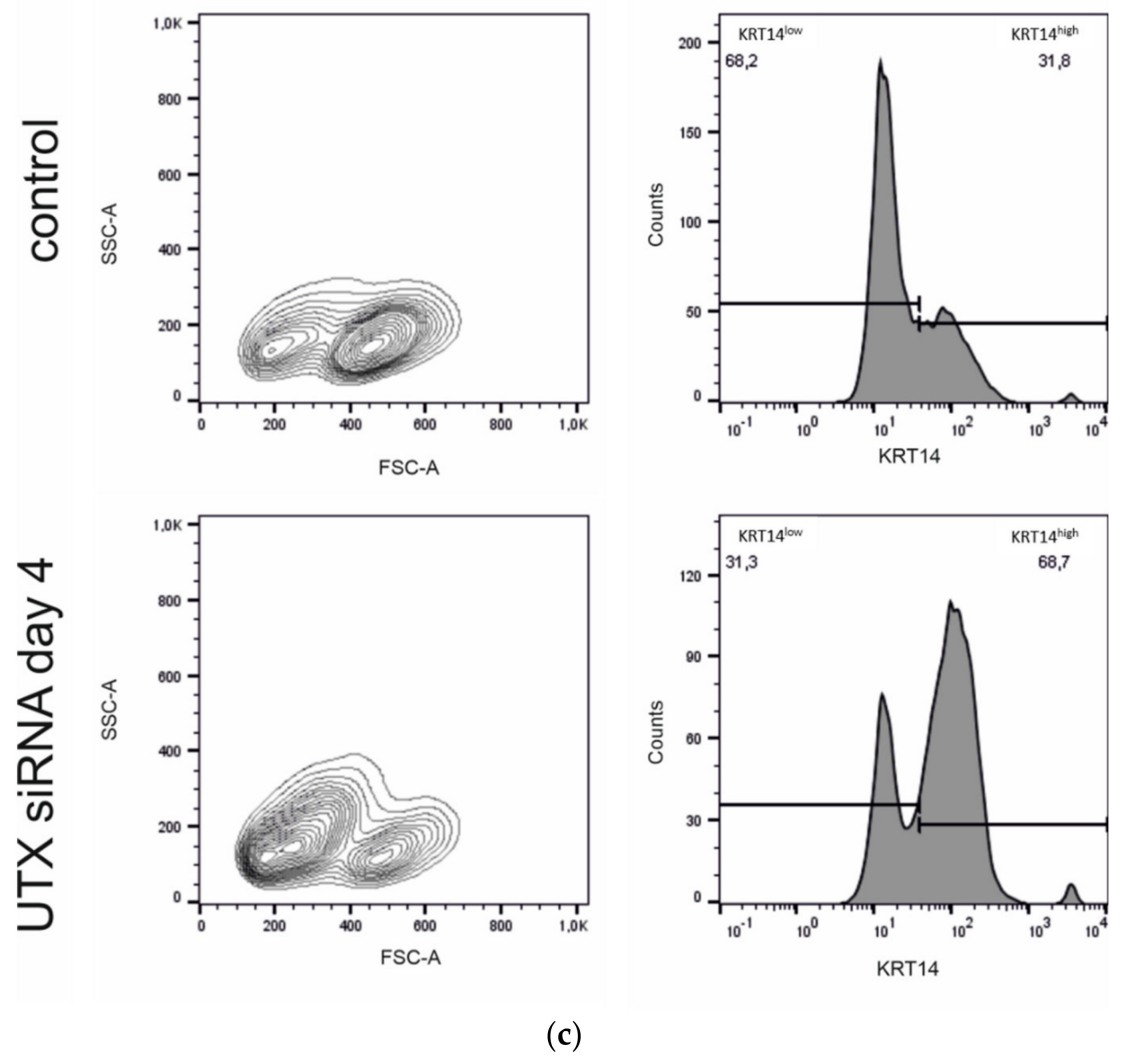
Enrichment of KRT14^high^ cells after UTX knockdown in HBLAK cells. (**a**) Immunocytochemical detection of KRT5 and KRT14, nuclei with DAPI and F-actin with phalloidin after transfection with UTX-siRNA for 2, 4 or 7 days. (**b**) Quantification of KRT14^high^ cells via a cell structure-related signal threshold analysis (ImageJ) in the HBLAK population following UTX knockdown. At least three experiments with >50 cells each were evaluated for each treatment and time point. The percentage of KRT14^high^ cells was measured via ImageJ analysis, as described in the Methods section. (**c**) KRT14 expression as detected by flow cytometry on day 4 after transfection of control siRNA or UTX-siRNA (*n* = 4). Statistics were performed with a two-way ANOVA with a post-hoc Tukey HSD test (## *p* < 0.01; ### *p* < 0.001).

As described above, following UTX knockdown, an increase of KRT14 mRNA was detected in both undifferentiated UEC and HBLAK cells (Figure 1b and Figure 2b). In HBLAK cells, this increase peaked around day 4, returning to control levels on day 7 (Figure 4). Analyses by immunocytochemistry (ICC) (Figure 4a and 4b) and flow cytometry (Figure 4c) revealed that the increase was in fact due to an increased proportion of KRT14^high^ cells. Two, four and seven days after transfection with UTX siRNA, without induction of differentiation, an increased fraction of KRT14^high^ cells was detected which likewise peaked at day 4 after knockdown (Figure 4). 

### 2.5. The HBLAK Cell Line Contains Two Major Subpopulations

**Figure 5:**
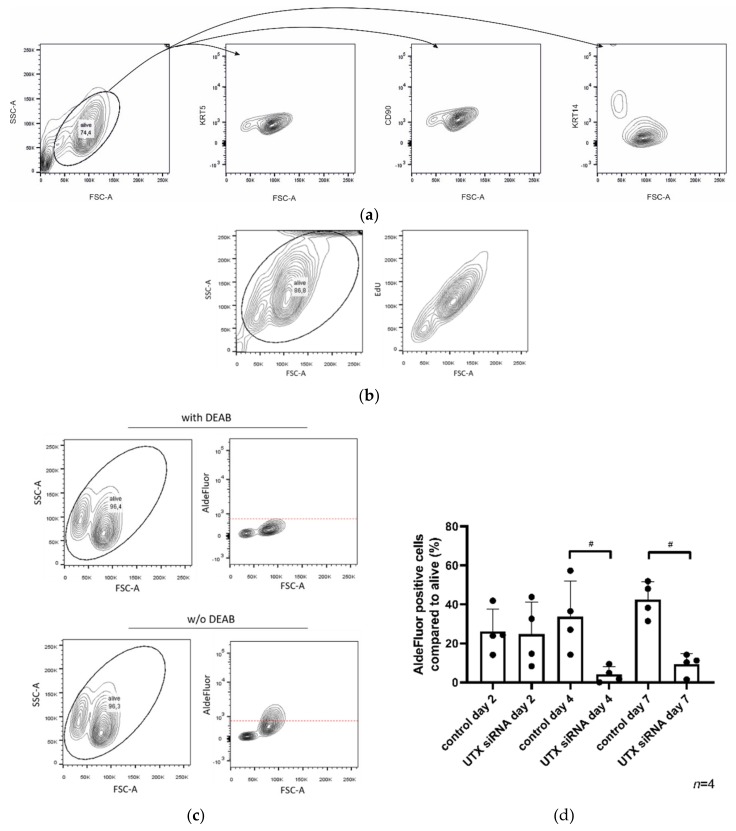
HBLAK contains two cell populations. (**a**) HBLAK cells homogeneously express CD90 and KRT5, but two subpopulations are distinguishable by size and KRT14 staining; namely, FSC-A^low^/KRT14^high^ and FSC-A^high^/KRT14^low^. (**b**) The larger KRT14^low^ cells are more intensely labeled by EdU staining. (**c**) The FSC-A^high^/KRT14^low^ population is additionally distinguishable by high AldeFluor-assay activity. In (**a**–**c**), evaluated cells are circled. (**d**) Effect of UTX knockdown using siRNA 01 on the proportion of the AldeFluor^pos^ population. As a negative control, DEAB reagent (inhibitor of the AldeFluor-assay) was used. Significant differences were observed 4 and 7 days after transfection of control siRNA or UTX-siRNA. Number of analyzed independent experiments, *n* = 4. Statistics were performed with a one-way ANOVA with a post-hoc Tukey HSD test (# *p* < 0.05).

To better understand the shift in the fraction of KRT14^high^ cells following UTX knockdown, the subpopulations present in HBLAK were characterized in more detail. FACS analysis allows one to distinguish two cell populations by size (Figure 5a), of which only the smaller cell population stains strongly for KRT14 (Figure 5a). EdU labeling revealed preferential EdU uptake into the larger KRT14^low^ cells (Figure 5b). The AldeFluor-assay likewise showed higher activity in the larger cell population (Figure 5c). These findings indicate that HBLAK contains two populations, namely, smaller, rather quiescent KRT14^high^/AldeFluor^low^ cells and larger, proliferating, KRT14^low^/AldeFluor^high^ cells. Staining for KRT5 or CD90 [16] did not distinguish these two populations in flow cytometry (Figure 5a). 

Following UTX knockdown, a strong shift towards the smaller-sized cell population can be observed in FACS analysis with a kinetics corresponding to the observations by ICC staining. Accordingly, the KRT14^high^/AldeFluor^low^ cells appear to increase in numbers, whereas KRT14^low^/AldeFluor^high^ cells decrease, in a dynamic manner (Figure 5d). As observed via other parameters (see Figure 4), the KRT14^low^/AldeFluor^high^ population was significantly diminished on day 4 after UTX knockdown but recovered thereafter.

**Figure 6:**
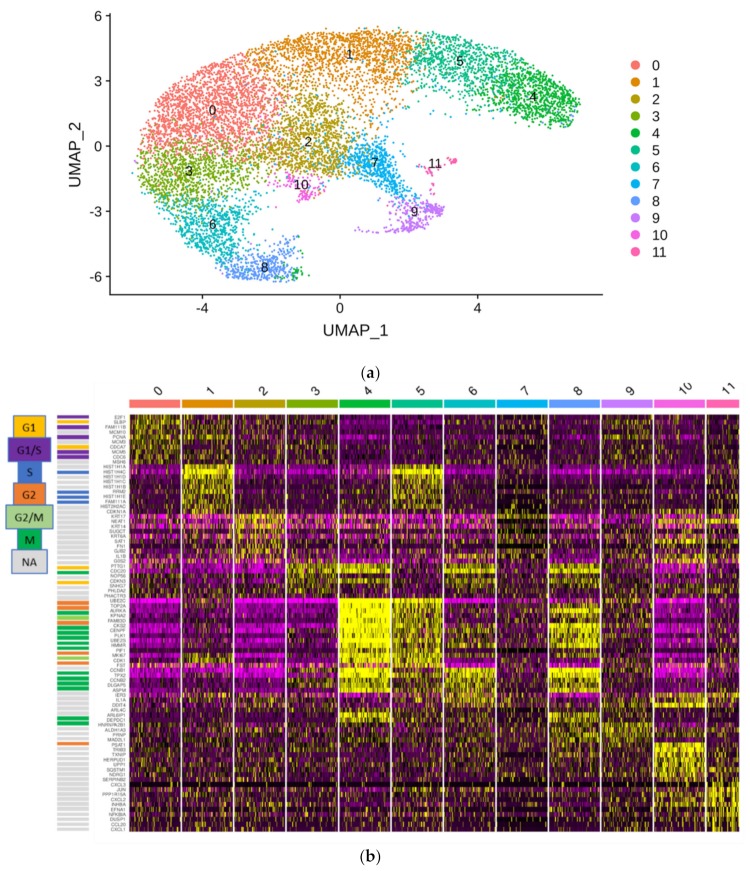
Single cell RNA-seq of the HBLAK cell line. Nearly 10,000 HBLAK cells were analyzed via single cell RNA-seq. (**a**) RNA-seq data analysis with the R toolkit “Seurat” (v3.0) and non-linear dimensional reduction using UMAP (Uniform Manifold Approximation and Projection) identifies 12 cell clusters. (**b**) Gene expression characteristics of the clusters. Expression levels range from purple (low) to yellow (high). Clusters are arranged from left to right; genes are listed on the left side of the main graph. The graphic on top highlights the distinctive gene functions in each cluster. Most clusters are distinguished by marker genes for different cell cycle phases; individual cell cycle genes, according to www.cyclebase.org, are color-coded as indicated in the legend on the very left: cluster 0—G1/S-phase; cluster 1—S-phase; cluster 3—G1-phase; cluster 4—G2-phase/mitosis; cluster 5—S-phase/G2-phase; cluster 6—G2-phase/mitosis; cluster 8—G2-phase/mitosis (UBE2C negative). Cluster 2 is distinguished by high KRT14 expression and lack of markers of active cell cycling. Smaller additional clusters are associated with reduced oxidative phosphorylation (cluster 7), increased regulation of cell death (cluster 9), increased stress response (cluster 10) and increased positive regulation of immune processes (cluster 11).

Analysis of the HBLAK cell line by single cell RNA sequencing (scRNA-seq) revealed several cell clusters (Figure 6a) including a KRT14^high^ cell cluster (cluster 2) which also expressed KRT5, KRT6A, KRT17 and SAT1 more strongly, whereas markers of an active cell cycle were expressed at low levels (Tables S1 and S2). In contrast, most other cell clusters (clusters 0, 1, 3–6, and 8) expressed KRT14 weakly but expressed active cell cycle markers. These clusters differed from each other, mostly by which cell cycle markers they expressed (Figure 6b). Additional smaller clusters were characterized by decreased oxidative phosphorylation (cluster 7), response to stress (cluster 9) and regulation of signal transduction (cluster 11). Cluster 10 was characterized by genes related to regulation of cell death and may represent dying cells. No clear correlation with specific clusters was observed for *ALDH* genes with the exception of *ALDH1A3*, which was more strongly expressed in clusters 2 and 9 (Figure 6b and Table S1). This analysis therefore demonstrates that KRT14^high^ cells are relatively quiescent compared to other subpopulations in the HBLAK cell line.

### 2.6. RNA-Seq Reveals Downregulation of Mitotic Pathways and Upregulation of p53-Signaling by UTX Knockdown

**Figure 7:**
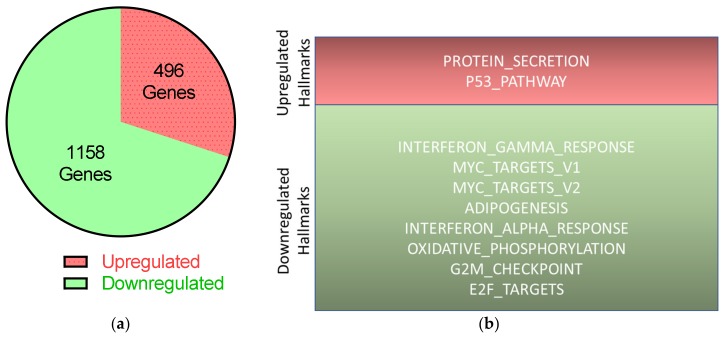
Transcriptional changes in HBLAK after UTX knockdown according to RNA-seq. On day two after transfection of control siRNA or UTX-siRNA 01, RNA was extracted from HBLAK cells and analyzed via RNA-seq. (**a**) Expressions of 496 genes were upregulated; 1158 genes were downregulated following UTX knockdown compared to transfection of control siRNA, with at least 1.5-fold changes and *p* < 0.05 after Bonferroni correction. (**b**) Results of further analyses of these significantly changed genes using the hallmark gene sets of the GSEA software (Molecular Signatures Database v7.0).

To understand how UTX knockdown might induce apoptosis in HBLAK cells, RNA-seq was performed using samples collected two days after UTX-siRNA transfection, i.e. before cell death peaks. Using Bonferroni correction and an >1.5 fold-change, 1158 genes were downregulated and 496 were upregulated (Figure 7, Table S3). Using the GSEA analysis tool [23], ten significantly altered gene groups (hallmarks) could be discerned of which eight were significantly downregulated. The two upregulated gene groups were “protein_secretion,” and intriguingly, “p53_pathway.” The most downregulated pathways were associated with mitosis, such as “E2F_Targets” and “G2M_Checkpoint,” in keeping with the observed decreased proliferation (Figure 3a). Gene ontology analysis using the STRING tool [24] additionally identified upregulation of genes involved in activation of apoptosis. According to the TFBS tool of the DAVID functional gene analysis [25,26], these genes were enriched in binding sites for p53, NFκB and FOXO1 (see Table S4).

### 2.7. Apoptosis Induced by UTX Knockdown is Partly Mediated by p53 Activation

**Figure 8:**
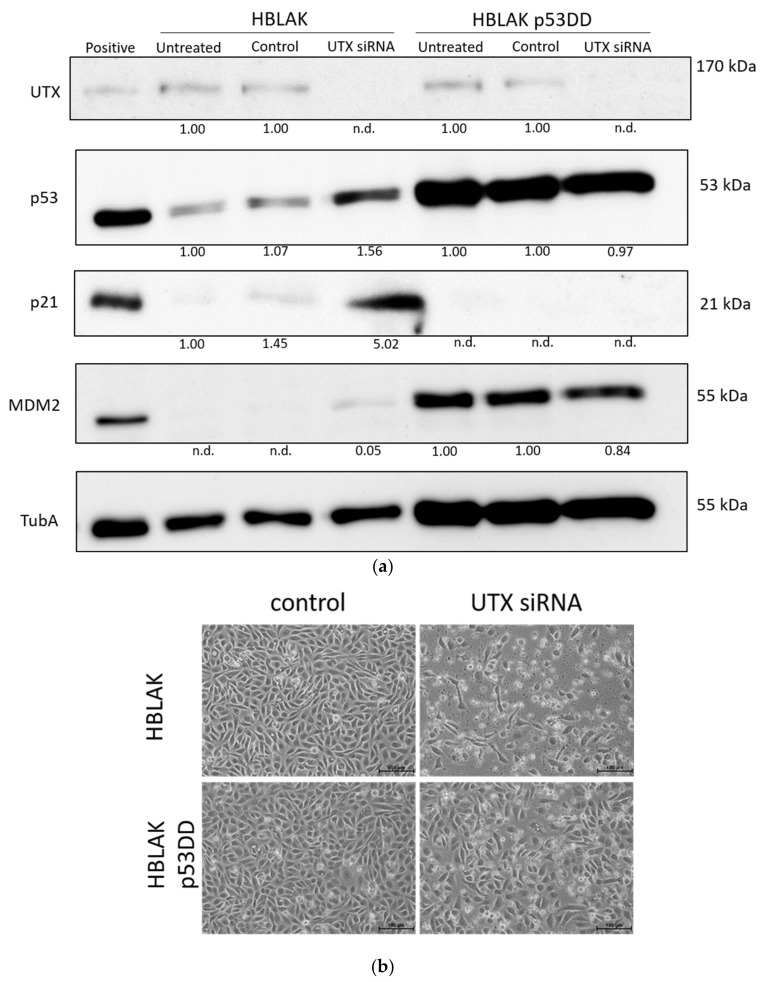

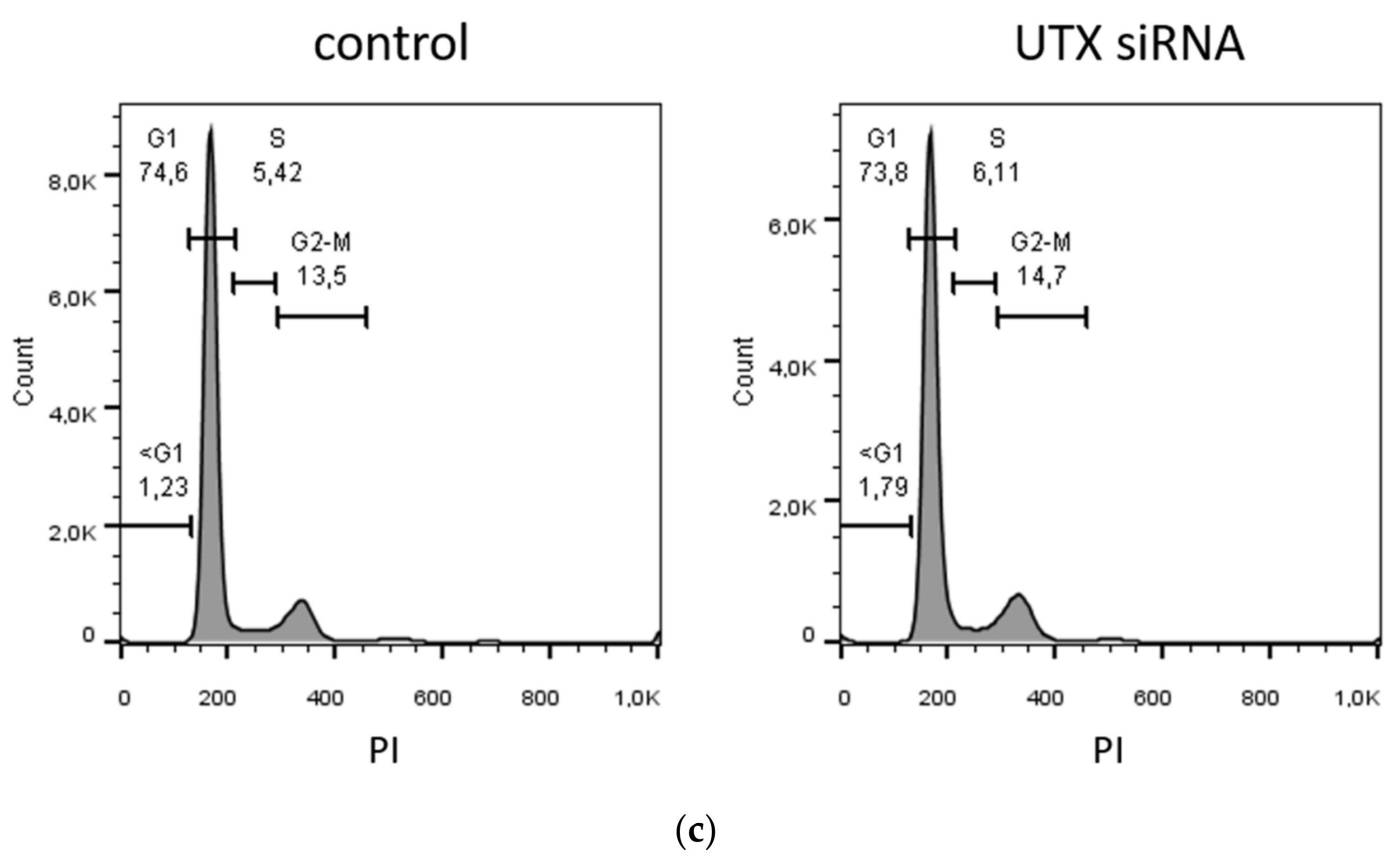
Involvement of p53 in apoptosis induction following UTX knockdown. (**a**) UTX, p53, p21 and MDM2 protein expression in wild-type HBLAK and dominant-negative p53 expressing HBLAK (HBLAK-p53DD) cells 48 h after transfection with UTX siRNA 01. α-Tubulin (TubA) was used as a loading control. As a positive control for p53, p21 and MDM2 expression, VM-CUB-1 cells were used. (**b**) Effects of UTX knockdown on morphology in wild-type HBLAK or HBLAK-p53DD cells 3 d after transfection. (**c**) Lack of changes in G1 and sub-G1 fractions in HBLAK-p53DD cells 2 days after UTX knockdown. Compare Figure 3C for wild-type cells. Number of analyzed experiments *n* = 4. The uncropped blots and molecular weight markers of (a) are shown in Figure S3.

According to RNA-seq analysis, several classical target genes of p53 are induced approximately two-fold (e.g., *CDKN1A*, *MDM2*, *FAS* and *TIGAR*) following treatment of HBLAK cells with UTX-siRNA. Accordingly, moderate increases in p53, p21 and MDM2 proteins could be observed by Western blot analysis (Figure 8). 

To investigate to which extent this moderate p53 increase was responsible for the observed cell death, we employed a HBLAK variant stably expressing a dominant-negative form of p53 (HBLAK-p53DD). In this cell line, p53 levels were enhanced, as expected for a cell line containing dominant-negative p53. Likewise, p21 levels appeared even lower than in parental cells, whereas MDM2 was increased. Neither protein was induced after treatment with UTX-siRNA. In these cells, death induced by UTX-siRNA was mitigated, but remained substantial (Figure 8). While further analyses will have to be performed to determine the overall effects of p53 inhibition on HBLAK cells, these findings support the involvement of p53 in the apoptosis induced by UTX knockdown. 

### 2.8. BFTC-905 Urothelial Carcinoma Cells Are Sensitive to UTX Knockdown

**Figure 9:**
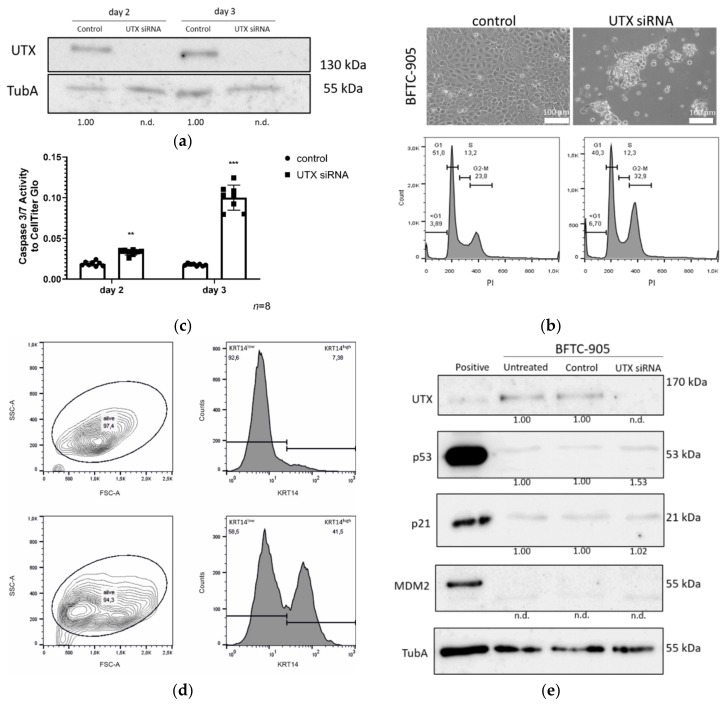
Effects of UTX knockdown on BFTC-905 urothelial carcinoma cells. (**a**) Efficient knockdown of UTX protein shown by Western blot analysis. (**b**) Changes in morphology, cell numbers and cell cycle profile on day 3 after transfection of UTX-siRNA 01 compared to control siRNA. Note that only a population of small cells normally located in the center of the colonies survives and the cell cycle profile shows increased fractions of cells in the G2/M and sub-G1 phases. (**c**) Caspase-Glo 3/7 activity relative to total viable cells measured by CellTiter-Glo assay 3 days after treatment with control siRNA or UTX-siRNA. (**d**) FACS analysis of cell size and KRT14 expression. Note the shift towards smaller cells in the FSC-A channel and an increased fraction of KRT14^high^ cells. Number of analyzed experiments *n* = 3. (**e**) UTX, p53, p21 and MDM2 protein expression in BFTC-905 cells 2 days after transfection with control siRNA or UTX-siRNA. α-Tubulin (TubA) was used as an internal loading control. As a positive control for p53, p21 and MDM2 expression VM-CUB-1 cells were used. Statistics were performed with a one sample *t* test against a set value of 1 (** *p* < 0.01; *** *p* < 0.001). The UTX siRNA knockdown samples at different time points were compared using an unpaired students *t* test. Scale 100 µm. The uncropped blots and molecular weight markers of (a) and (e) are shown in Figures S4 and S5.

We have previously observed that the urothelial carcinoma cell line BFTC-905, with an overall basal phenotype similar to those of UECs and HBLAK, harbors a possible stem cell-like population [27]. Efficient knockdown of UTX in this cell line likewise elicited a strong apoptotic effect after 2–3 days (Figure 9a–c). These cells also contain a KRT14^high^ subpopulation, which, like in HBLAK and UEC cells, increased after UTX knockdown, as observed via flow cytometry (Figure 9d) and immunocytochemistry [28]. Cell cycle profiles revealed increased G2/M and sub-G1 fractions following UTX knockdown already two days after siRNA transfection (Figure 9b). Unlike in HBLAK cells, p53 protein and its targets p21 and MDM2 were not increased (Figure 9e) in the BFTC-905 cell line, which expresses low basal levels of all three proteins. AldeFluor activity was not associated with any specific subpopulation under basal growth conditions and accordingly, did not significantly shift following UTX-siRNA treatment [28].

## 3. Discussion

Mutations inactivating UTX are found across all stages of urothelial carcinoma (UC), albeit more commonly in lower stage tumors [11], and intriguingly, across all molecular subtypes of muscle-invasive bladder cancers (MIBC) [2]. In particular, according to the large TCGA study [10], there is no significant difference in their frequency between cancers with a basal-squamous (BASQ) phenotype, which present with markers of basal urothelial cells, and cancers with luminal phenotypes exhibiting markers of intermediate and luminal differentiated urothelial cells. Most current evidence indicates that basal cells are precursors of intermediate and luminal cells [12,13,14,16,17]. Therefore, it may seem a priori unlikely that UTX inactivation contributes to urothelial carcinogenesis by blocking the differentiation of basal to luminal cells, since in that case UTX mutations should be more prevalent in BASQ UC. This assumption is borne out by our finding that UTX knockdown in models of urothelial differentiation, wherein cells with a basal phenotype differentiate into luminal cells, did not significantly inhibit differentiation. Our conclusion should, however, be considered with caution in so far, as in vitro differentiation models of urothelial cells do not in every respect fully recapitulate in vivo biochemical differentiation and stratification. 

Our investigation focused on an unexpected observation; namely, that the UTX knockdown elicited significant cell death by apoptosis. This occurred independent of differentiation treatment after 2–3 days in cultured normal urothelial cells (UECs) and immortalized HBLAK cells. A similar effect was elicited in the BFTC-905 UC cell line, which like the other two cell types has a basal phenotype [27,29]. All three models moreover share the properties of being wild-type for p53 and all COMPASS components and containing a fraction of cells with high expression of KRT14, in addition to the ubiquitously expressed basal cell cytokeratin KRT5. KRT14 is generally considered a marker of urothelial stem cells [12,13,14,15,16,17]. In the urothelium between 1% and 14% of basal urothelial cells are estimated as KRT14^high^, depending on developmental stage, physiological state and species [17]. Following UTX knockdown, we observed a relative increase of KRT14 expression in the urothelial culture models, which upon closer analysis turned out to reflect an increased fraction of KRT14^high^ cells due to apoptotic death of KRT14^low^ cells.

Since UECs are not consistently available and can be highly variable between individual cultures, we investigated this phenomenon in depth in HBLAK, and subsequently, in BFTC-905 cells. The previously reported KRT14^high^ fraction in HBLAK turned out to differ from KRT14^low^ cells by further properties; namely, cell size, proliferative activity and AldeFluor-assay activity. The observations that KRT14^high^ cells are smaller and less proliferative than KRT14^low^ cells are in accordance with expectations for a stem cell population. AldeFluor-assay activity would instead be expected to be higher in stem cells, oppositely to our observations. In fact, AldeFluor-assay activity reflects the activity of various aldehyde dehydrogenases [30], whose expression does not necessarily have to be localized in the stem cell population of a tissue. AldeFluor activity was linked to tumor-initiating cells in bladder cancer in a previous publication, but the authors did not investigate the association with KRT14 expression [31]. In a survey of three different UC cell lines (T24, TCCSUP and 5637), however, AldeFluor activity was not consistently associated with stem cell properties [32]. Likewise, the lack of association between AldeFluor-assay activity and subpopulations in BFTC-905 argues against a strict link between stem cell properties and AldeFluor activity in urothelial cancer. Our results, rather, suggest that high ALDH activity may characterize KRT5-positive basal cells in the urothelium, possibly due to the most prominently expressed ALDH1A3 isoenzyme. Regardless, UTX knockdown enriched for the small cell, KRT14^high^, weakly proliferative and AldeFluor^low^ cell population in HBLAK cells. 

Collectively, these findings suggest that loss of UTX favors the survival of urothelial KRT14^high^ stem cells over more differentiated KRT14^low^/KRT5+ basal cells. In vivo, loss of UTX could therefore over time lead to an expansion of the stem cell population with an increased likelihood of transformation. Notably, a function of UTX in the regulation of the ratio of stem cells to more differentiated basal cells rather than during further urothelial differentiation would account for the prevalence of UTX mutations throughout all UC subtypes. This argument also suggests that UTX inactivation might constitute an early event in urothelial carcinogenesis. Additional mutations in p53 or growth factor receptors would then lead to cancer. For instance, activation of STAT3 in a mouse model of urothelial carcinoma was associated with expansion of KRT14^high^ cells [33]. Urothelial cancer is notorious for its multifocality and high recurrence rate after local surgery, which points towards a pronounced field effect. Recent studies have indeed observed clonal expansion of mutant cells in morphologically normal urothelium. In one study on four patients, intriguingly, *KMT2D*, another COMPASS component, was frequently mutated in morphologically normal urothelial tissue from cancer-carrying bladders [34]. An analogous scenario is established in the development of acute myeloid leukemia, which is often preceded by clonal hematopoiesis elicited by mutations in various genes, most often encoding epigenetic regulators like DNMT3A and TET2 [35], which shift the balance between stem cells and differentiated progeny and displace normal with stem cells with mutants. Similarly, mutations inactivating *KMT2D* appear to increase the B-cell population at risk to develop lymphomas by further genetic alterations [36]. 

Cell death after UTX knockdown in HBLAK was partly mediated by p53, as suggested by the induction of canonical p53 response genes and by the decrease in cell death in HBLAK cells expressing a dominant-negative p53 protein. In the context of urothelial carcinogenesis, this may be relevant, as p53 function is obliterated or impeded in most MIBC. The cells most affected by UTX knockdown were the proliferating KRT14^low^ cells. Our findings might therefore predict that in vivo UTX-mutant cells might be able to proliferate identically or better if p53 is also inactivated. In other words, UTX mutations may select for additional genetic changes that inactivate p53. However, expression of a dominant-negative p53 protein only diminished, but did not obliterate cell death in HBLAK cells treated with UTX-siRNA, indicating the involvement of additional factors. Deeper analysis of the RNA-seq data suggests activation of FOXO transcription factors, which can also induce apoptosis, as a second possible factor. It is instructive to compare this situation to the regenerating liver, where, likewise, quiescent cells re-enter a proliferative state. Here, fine-tuning of the activity of p53 and further transcription factors by several pathways is required to avoid induction of apoptosis during proliferation and ensure genetic stability and correct lineage choice [37]. In the urothelium, another tissue with high regenerative potential, such mechanisms have not yet been sufficiently studied. It should also be mentioned that cell death in BFTC-905 cells did not appear to be associated with p53 activation at all. Another difference in the BFTC-905 UC cell line compared to HBLAK cells was a clearly increased fraction of G2/M-phase cells. This further indicates that the response to UTX knockdown likewise lead to an enrichment of KRT14^high^ cells, but differed somewhat from that in HBLAK. These differences underline the conclusion that additional pathways beyond p53 activation are involved in the response to UTX knockdown.

Our findings raise the question of why UTX is required for survival of proliferating KRT14^low^ cells. The most likely answer is that these cells require UTX to set up a new stable epigenetic state. In support of this idea, the UTX antagonist EZH2 has been shown to be required for proper regeneration of the urothelium following damage by uropathogenic bacteria [38]. The function of UTX during urothelial regeneration, which involves repletion of intermediate luminal, and especially umbrella cells, from the lower epithelial layers, should therefore be studied. Since interactions of UTX with a broad range of lineage-specific transcription factors have been described (reviewed in [2]), another not necessarily exclusive explanation is that UTX may be required as a co-activator for specific transcription factors establishing the epigenetic state of KRT14^low^/KRT5+ cells. These issues will require further experimental investigation.

## 4. Materials and Methods 

### 4.1. Cell Culture

HBLAK cells were obtained from CELLnTEC Advanced Cell Systems (Bern, Switzerland) and were routinely cultured in CnT-Prime (CnT-PR), as described in [20]. UECs were cultured in KSFM supplemented with epidermal growth factor and bovine pituitary extract, as described in [39]. Both were passaged using accutase (Sigma Aldrich, Munich, Germany). Culture and use of normal urothelial cells from ureters of patients undergoing nephrectomy was permitted by the ethical committee of the HHU medical faculty (#1788). UC cell lines were cultured in Dulbecco’s modified eagle’s medium (DMEM, ThermoFisher Scientific, Langenselbold, Germany) supplemented with 10% fetal calf serum as previously described [21]. Passaging was performed using trypsin (Sigma Aldrich, Munich). All cells were cultured at 37 °C with 5% CO_2_. All cells were regularly authenticated by STR profiling and checked for mycoplasm contamination.

The SW1710-UTX-KO cell line was generated using UTX CRISPR/Cas9 KO Plasmids (Santa Cruz Biotechnology, Dallas, TX, USA, sc-402761-NIC and sc-402761-NIC-2) and clonal selection with 0.5 µg/mL puromycin (Invivogen, Toulouse, France) for 5 days as described [21]. 

To generate HBLAK-p53DD cells, HBLAK cells were infected with the retroviral vector pBABE-hygro-p53DD (Addgene plasmid #9058, kindly provided by Prof. R. Weinberg, Boston, MA, USA). This vector expresses p53DD, a dominant-negative p53 mutant, consisting of the initial 14 amino acids and the oligomerization and COOH-domains of p53 but lacking the intermediate 288 (15–301) amino acids [40]. Transduced cells were then selected with hygromycin.

### 4.2. Induction of Urothelial Differentiation 

Two days after siRNA transfection, HBLAK cells were switched to CnT-PR-D medium (CELLnTEC) and treated with 2 mM CaCl_2_ and 5% fetal calf serum (Ca/FCS) and UECs were treated with 1 µM troglitazone and 1 µM PD153035 (TZ/PD) and further cultured for up to 10 days. 

### 4.3. siRNA Transfection

All siRNAs were transfected at a final concentration of 10 nM using Lipofectamine™ RNAiMAX Transfection Reagent (ThermoFisher Scientific, catalogue number 13778150). The following siRNAs were purchased from ThermoFisher Scientific: ON-TARGETplus Human KDM6A siRNA (SMARTpool, Dharmacon, L-014140-01-0005, siRNA 01) with ON-TARGETplus Non-targeting Pool (Dharmacon, D-001810-10-05, siRNA 20) as a control, or Silencer® Select siRNA (4392420, Ambion, KDM6A, s14735) with Silencer™ Select Negative Control No. 1 siRNA (Invitrogen, 4390843, Waltham, MA, USA). 

### 4.4. RNA isolation and RT-qPCR

Cells were lysed with TRIzol™ Reagent (Invitrogen, 15596026). Following addition of chloroform, RNA, DNA and protein were separated via centrifugation at 11,000 g at 4 °C for 10 min. RNA was then isolated by adding one volume of EtOH (70%) and further purified using the RNeasy Mini Kit (Qiagen, Hilden, Germany, catalogue number 74106). For reverse transcription 1 µg RNA was copied into cDNA using the QuantiTect Reverse Transcription Kit (Qiagen, catalogue number 205313). qPCR was performed with the QuantiTect SYBR Green PCR (Qiagen catalogue number 204145). The following primers were used: 

KRT14 (forward: GCG CAC CAT GCA GAA CCT G reverse: CCT CCA CGC TGC CAA TCA TC, at 55 °C) 

KRT20 (forward: GAA GTC CTC AGC AGC CAG TT, reverse: GGT CGC GAC TAC AGT GCA TA, at 60 °C) 

UPK2 (forward: GAC AGC CAC TGA GTC CAG CAG, reverse: AGC ACC GTG ATG ACC ACC ATG, at 60 °C), 

UTX (forward: CGA AAA ACA AGC GGA AAC T, reverse: TAT CAA GAT GAG GCG GAT G, at 55 °C), 

TBP (forward: ACA ACA GCC TGC CAC CTT A, reverse: GAA TAG GCT GTG GGG TCA GT, at 60 °C)

Standard curves were carried in each experiment to calculate relative expression, and *TBP* was used as a reference gene.

### 4.5. Immunocytochemistry (ICC)

Cells were fixed and permeabilized using a solution of 1% paraformaldehyde and 0.02% Triton X-100 in PBS. All antibodies were diluted in the blocking solution (1% BSA, 0.1% saponine, 0.1% NaN_3_, dissolved in PBS). Used antibodies were: KRT5 (Abcam, Cambridge, UK, ab53121, at a 1:250 dilution) and KRT14 (Abcam, ab7800, 1:250) with secondary antibodies goat-anti-mouse IgG Alexa Fluor 633 (ThermoFischer Scientific, A-21052) and goat-anti-rabbit IgG Alexa Fluor 488 (ThermoFischer Scientific, A-11008). Phalloidin (Acti-stain 535 Phalloidin, tebu-bio, PHDR1, 1:1,000) was used to stain actin filaments and 4′,6-diamidine-2′-phenylindole dihydrochloride (DAPI, Roche, Mannheim, Germany, 10236276001) to stain DNA. Cover slips were mounted with Dako Fluorescence Mounting Medium (Dako, S3023). Detection was performed using ZEISS Axio Observer.Z1/7; Plan-Apochromat 40× /1.4 Oil DIC (UV) VIS-IR M27; 90 HE DAPI/ GFP/ Cy3 /Cy5; LED-module "wavelength" nm (Colibri 7); ZEISS Axiocam 512 mono.

Pictures were analyzed with ImageJ (version 1.52b) and a specific approach-based macro. The core adjustments were: DAPI-Channel (threshold: "Default"; "Watershed"); KRT14-Channel (threshold: "Huang"; "Watershed"). Regions of interest were counted by using "Extended Particle Analyzer" with a pixel ratio of 1:100,000.

### 4.6. Western Blot Analysis 

Cells were scraped off, washed once with PBS and collected by centrifugation at 500 g for 5 min at RT. The cell pellet was then lysed with NP-40 lysis buffer (150 mM NaCl, 1% NP-40, 0.1% SDS, 1 mM EDTA, 50 mM Tris-HCl, pH 7.6, with protease inhibitor (Sigma Aldrich, P8340)). Lysates were cleared by centrifugation at 11,000 g for 20 min at 4 °C. Protein concentration was measured by the Pierce™ BCA Protein Assay Kit (Sigma Aldrich, 23225). Western blotting was performed using 15–50 µg protein in 10% or 12% SDS-PAGE gels in TGS-buffer (Bio-Rad, Feldkirchen, Germany, 1610732) at 20 mA for 60 min followed by transfer (100 V for 40 min) to a PVDF membrane with blotting buffer (125 mM Tris, 960 mM glycine, 10% methanol). The membrane was blocked with 5% milk in TBS-Tween (0.1%). The following antibodies were applied in TBS-Tween (0.1%); TubA (Abcam, #ab4074, at a dilution of 1:10,000); UTX (Cell Signal Technology, Frankfurt, Germany, 33510, 1:500); p53 (Merck Millipore, #OP43, at a dilution of 1:500); p21 (Merck Millipore; Darmstadt, Germany #OP64, at a dilution of 1:500); MDM2 (Oncogene Science, Uniondale, USA, #OP46, at a dilution of 1:500); goat anti-rabbit IgG H&L (HRP, Abcam, ab6721, 1:10,000); H3K27me2/3 (Active Motif, La Hulpe, Belgium, #39535); H3 (Cell Signaling Technology, #4499); goat anti-rabbit IgG H&L (HRP, Dako, Glostrup, Denmark, #P0448, 1:1,000); rabbit anti-mouse IgG H&L (HRP, Dako, #P0260, 1:1,000); IRDye^®^ 800CW goat anti-rabbit IgG (LI-COR Biosciences, Lincoln, NE, USA, 925-32211, 1:20,000). Membranes were developed by Clarity Western ECL Substrate (Bio-Rad #170-5061), and detection was performed on a Chemidoc Imagine System (Bio-Rad) or an Odyssey Classic with Image Studio Software (version 4.0, LI-COR Biosciences, Lincoln, NE, USA). Quantification as indicated below each blot figure was performed relative to tubulin α or histone H3 using the respective instrument or ImageJ software [41].

### 4.7. Flow Cytometry

Cells were cultured on six-well plates and harvested by accutase or trypsin. After one washing, step cells were fixed and permeabilized using a solution of 1% paraformaldehyde and 0.02% triton X-100 in PBS. Antibodies against KRT5 (Sigma Aldrich, FCMAB291F, dilution 1:200), CD90 (Miltenyi Biotech, Bergisch-Gladbach, Germany, 120-007-297, dilution 1:100) and KRT14 (Novus Biologicals, Abingdon, UK, NBP2-34403APC, dilution 1:100), were diluted in blocking solution (1% BSA, 0.1% saponin, 0.1% NaN_3_ in PBS). Negative controls were performed with mouse Anti-IgG1-APC (Miltenyi Biotech, 130-117-058), mouse Anti-IgG1-PE (Miltenyi Biotech, 130-117-057) and mouse Anti-IgG1-FITC (Miltenyi Biotech, 130-095-897).

### 4.8. Viability Assay

Two-thousand cells in 100 µL cell culture media were seeded per well in 96-well-plates. After one day of culture cells were transfected with UTX or control siRNA. Two days later, viable cells were quantified by addition of 10 µL MTT for one or four (HBLAK) hours. After incubation at cell culture conditions, the supernatant was removed and cells were lysed in 50 µL DMSO. Absorbance was measured at 505 nm.

### 4.9. EdU-labeling

Cells were cultured and treated on six-well plates. On the day of measurement, the cells were incubated with 10 μM EdU for 4 h. Fixation and further steps were performed as described by the manufacturer (Sigma Aldrich, BCK-FC647-100). Detection was performed using a Miltenyi MACSQuant^®^ Analyzer (Milteny Biotec, Bergisch-Gladbach, Germany) and evaluated using FlowJo (BD, Version 10.4, Franklin Lakes, NJ, USA).

### 4.10. AldeFluor-Assay

Cells were cultured and transfected on six-well plates. After harvesting and washing once with PBS, cells were resuspended in the AldeFluor reaction solution, as described by the manufacturer (STEMCELL Technologies, Cologne, Germany, catalogue number #01700). The detection was performed on a Miltenyi MACSQuant^®^ Analyzer (Milteny Biotec) and evaluated using FlowJo (BD, Version 10.4, Franklin Lakes, NJ, USA).

### 4.11. Cell Cycle Analysis by Flow Cytometry

Cell cycle analyses were performed as previously described [42]. Soluble and floating cells collected from the supernatant were stained with Nicoletti-buffer (50 µg/µL propidium iodide (PI), 0.1% sodium citrate and 0.1% Triton X-100), and profiles were established using a Miltenyi MACSQuant^®^ Analyzer (Milteny Biotec) and evaluated using FlowJo (BD, Version 10.4 Franklin Lakes, NJ, USA).

### 4.12. Caspase and CellTiter-Glo Assay

After one day of culture following siRNA transfection in 6-well plates, 2000 cells in 50 µL cell culture media were seeded on 96-well plates. After further two days of culture, 50 µL of Caspase-Glo^®^ Reagent (Promega, G8090, Madison, WI, USA) or CellTiter-Glo^®^ (Promega, G7570) was added to each well. After 30 min of incubation at 37 °C, signals were detected as described by the manufacturer.

### 4.13. RNA-Seq

Total RNA samples used for transcriptome analyses were quantified (Qubit RNA HS Assay, Thermo Fisher Scientific) and quality measured by capillary electrophoresis using the Fragment Analyzer and the Total RNA Standard Sensitivity Assay (Agilent Technologies, Inc. Santa Clara, CA, USA). All samples showed high quality RNA quality numbers (≥10). The library preparation was performed according to the manufacturer’s protocol using the “TruSeq Stranded mRNA Library Prep Kit” from Illumina^®^. Briefly, 300 ng total RNA were used for mRNA capturing, fragmentation, the synthesis of cDNA, adapter ligation and library amplification. Bead purified libraries were normalized and finally sequenced on the HiSeq 3000 system (Illumina Inc. San Diego, CA, USA) with a read setup of 1 × 150 bp. The bcl2fastq tool was used to convert the bcl files to fastq files as well for adapter trimming and demultiplexing. 

Data analyses on fastq files were conducted with CLC Genomics Workbench (version 10.1.1, QIAGEN, Venlo, The Netherlands). The reads of all probes were adapter trimmed (Illumina TruSeq) and quality trimmed (using the default parameters: bases below Q13 were trimmed from the end of the reads, ambiguous nucleotides maximal 2). Mapping was done against the Homo sapiens (hg38) (Mai 25, 2017) genome sequence. After grouping of samples (three biological replicates each), a pairwise comparison was made and statistically determined using the Empirical Analysis of DGE (version 1.1, cutoff = 5). The Resulting *p* values were corrected for multiple testing by FDR and Bonferroni-correction. A *p* value of ≤0.05 was considered significant. RNA-seq data were further analyzed via the GSEA Software (GSEA v4.0.1 for Windows) [43], the String (https://string-db.org/) [24] and DAVID databases [25,26]. The RNA-seq and the single cell RNA-seq data are available through the GEO database.

### 4.14. Single Cell RNA-Seq

HBLAK cells were harvested by accutase after routine cultivation in T25 flasks for two days. Cell viability and cell number analysis were performed via trypan blue staining in a Neubauer counting chamber. A total of 20,000 cells were used as input for the single-cell droplet libraries generation on the 10× Chromium Controller system utilizing the Chromium Single Cell 3 Reagent Kit v3 according to manufacturer’s instructions. Sequencing was carried out on a HiSeq 3000 system (Illumina Inc. San Diego, CA, USA) with a mean sequencing depth of ~40,000 reads/cell. Raw sequencing data were processed using the 10× Genomics CellRanger software (v3.1). Raw BCL-files were demultiplexed and processed to Fastq-files using the CellRanger mkfastq pipeline. Alignment of reads to the mm10 genome and UMI counting was performed via the CellRanger count pipeline to generate a gene-barcode matrix.

Further analyses were carried out with the Seurat v3.0 R package. Initial quality control consisted of removal of cells with fewer than 200 detected genes and removal of genes expressed in fewer than 3 cells. Furthermore, cells with a mapping rate of >10% to the mitochondrial genome were removed, under the assumption that they represent dead or damaged cells. Normalization was carried out utilizing SCTransform. Dimensional reduction of the data set was achieved by principal component analysis (PCA) based on identified variable genes and subsequent UMAP embedding. The number of meaningful principal components (PC) was selected by ranking them according to the percentage of variance explained by each PC, plotting them in an “elbow plot” and manually determining the number of PCs that represent the majority of variance in the data set. Cells were clustered using the graph-based clustering approach implemented in Seurat v3.0. Markers defining each cluster and differential gene expression between different clusters were calculated using a Wilcoxon Rank Sum test which was implemented in Seurat.

### 4.15. Statistics

All data were analyzed using the IBM SPSS Statistics v26.001 for Windows (2019) program. Results with *p* ≤ 0.05 were considered significant. Differences in mean values between two groups were statistically confirmed by means of the *t* test for independent samples. In all parametric tests used, the respective requirements were checked, and appropriate consequences were drawn for events of gross injuries. Unless indicated otherwise, the Tukey test was used as a post-hoc test to identify group differences in variance analysis. 

## 5. Conclusions

In conclusion, we report that knockdown of UTX, while not severely impeding urothelial differentiation, induces apoptosis in urothelial cell models that lack mutations in COMPASS complex genes. Our data suggest that UTX is particularly important for the survival of proliferating cells that have exited the more quiescent stem cell compartment marked by high expression of KRT14. UTX loss, therefore, shifts the balance towards KRT14^high^ cells. This hitherto undescribed and unexpected function may help to explain why mutations inactivating UTX are prevalent throughout out all stages and subtypes of UC. We suggest that loss of UTX function may promote the expansion of clonal cell populations in the urothelium that can generate tumors after acquiring additional mutations inactivating tumor suppressors, such as p53, or activating oncogenes, such as FGFR3.

## Supplementary Materials

The following are available online at https://www.mdpi.com/2072-6694/12/4/1023/s1. Figure S1: Modulation of KDM6A/UTX expression in human urothelial carcinoma cells, Figure S2: Two different UTX siRNA pools similarly reduce the viability of HBLAK cells 4 d after transfection, Figure S3: The uncropped blots and molecular weight markers of Figure 8a, Figure S4: The uncropped blots and molecular weight markers of Figure 9a, Figure S5: The uncropped blots and molecular weight markers of Figure 9e, Table S1: Gene markers of all cell clusters of the single cell RNA-seq experiment generated via the Seurat R Package, Table S2: Average gene expression of all cell clusters of the single cell RNA-seq experiment generated via the Seurat R Package, Table S3: RNA-seq data table containing the analysis of the HBLAK RNA-seq experiment 2 d after UTX siRNA transfection, Table S4: UCSC-TFBS analysis of upregulated apoptosis-related genes in HBLAK cells 2 d after UTX siRNA transfection.
