# Supplementary figures and images for: Knockdown of UTX/KDM6A Enriches Precursor Cell Populations in Urothelial Cell Cultures and Cell Lines
Source: Cancers (Basel). 2020 Apr 21;12(4):1023. doi: 10.3390/cancers12041023 (PMC7226239; doi:10.3390/cancers12041023)

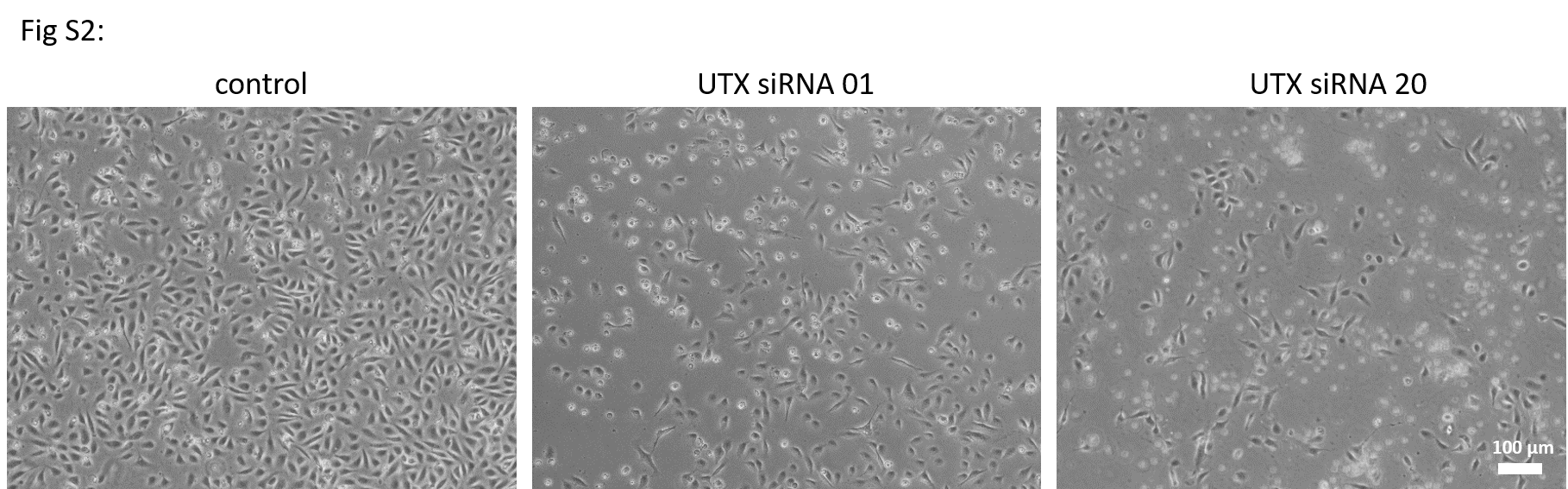

Supplement: Supplementary file 1 [file cancers-12-01023-s001.zip › Fig S2.tif]

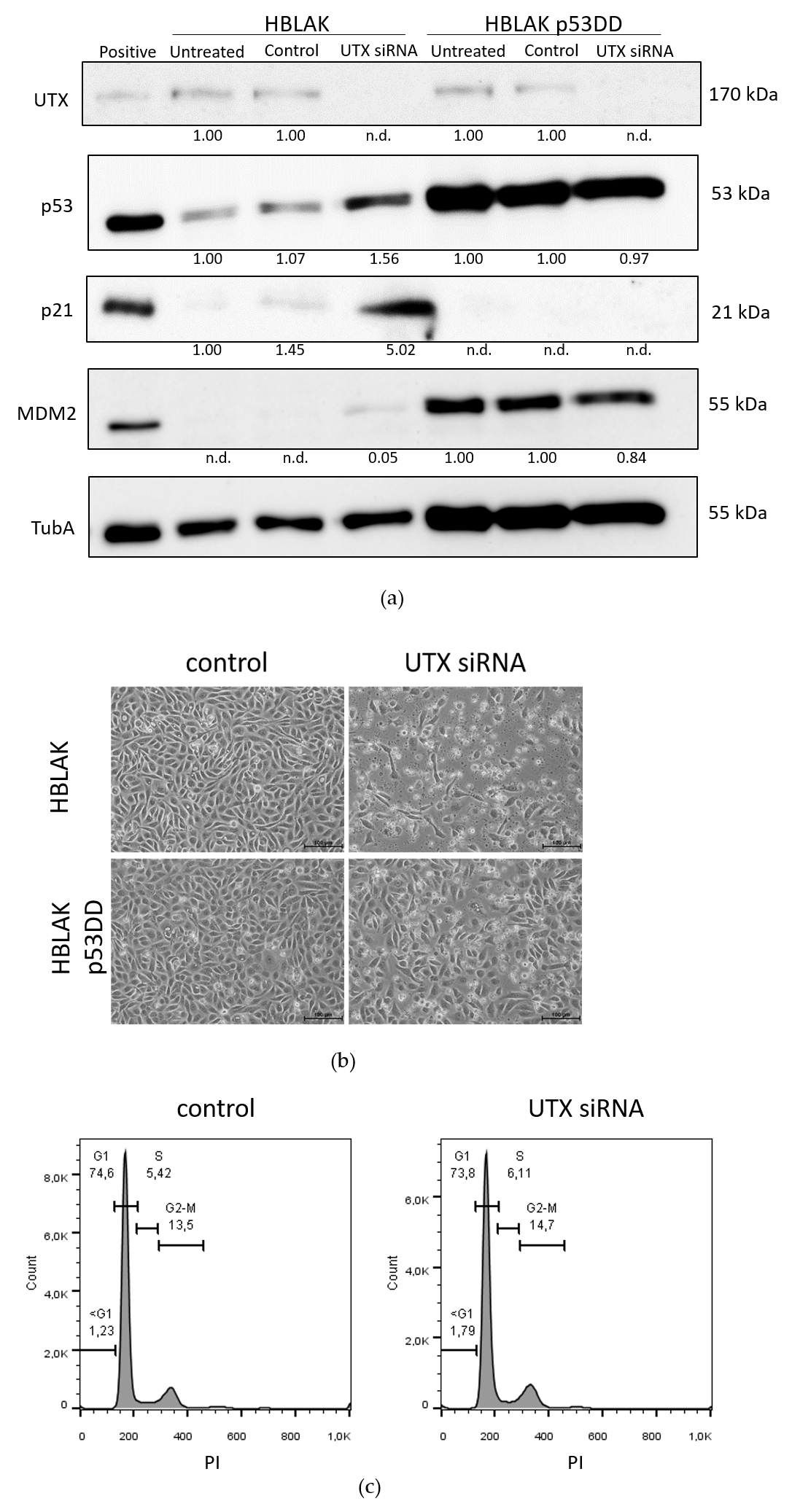

Supplement: Supplementary file 1 [file cancers-12-01023-s001.zip › Western Blots/Fig.8n.tif]

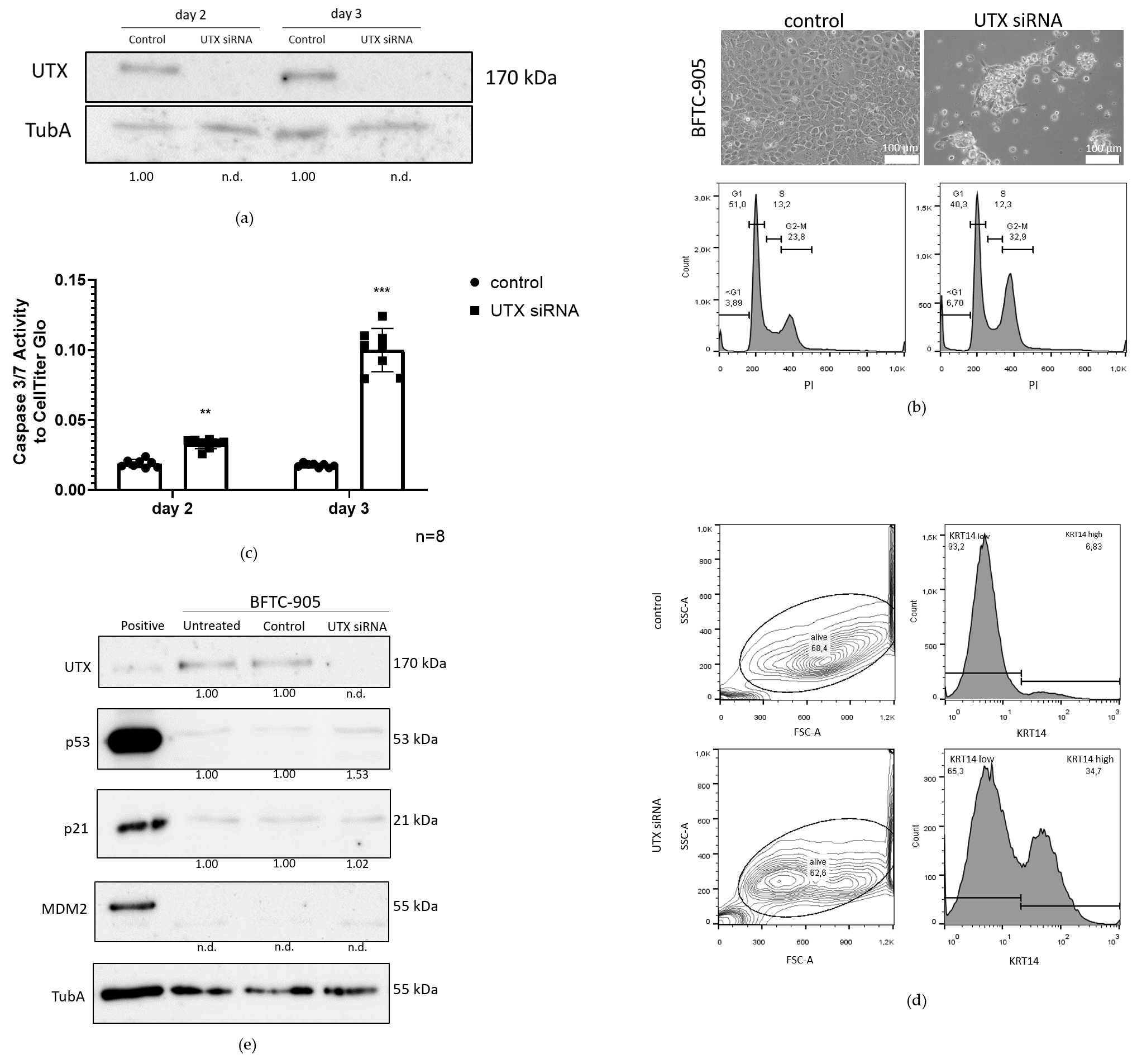

Supplement: Supplementary file 1 [file cancers-12-01023-s001.zip › Western Blots/Fig.9n.tif]

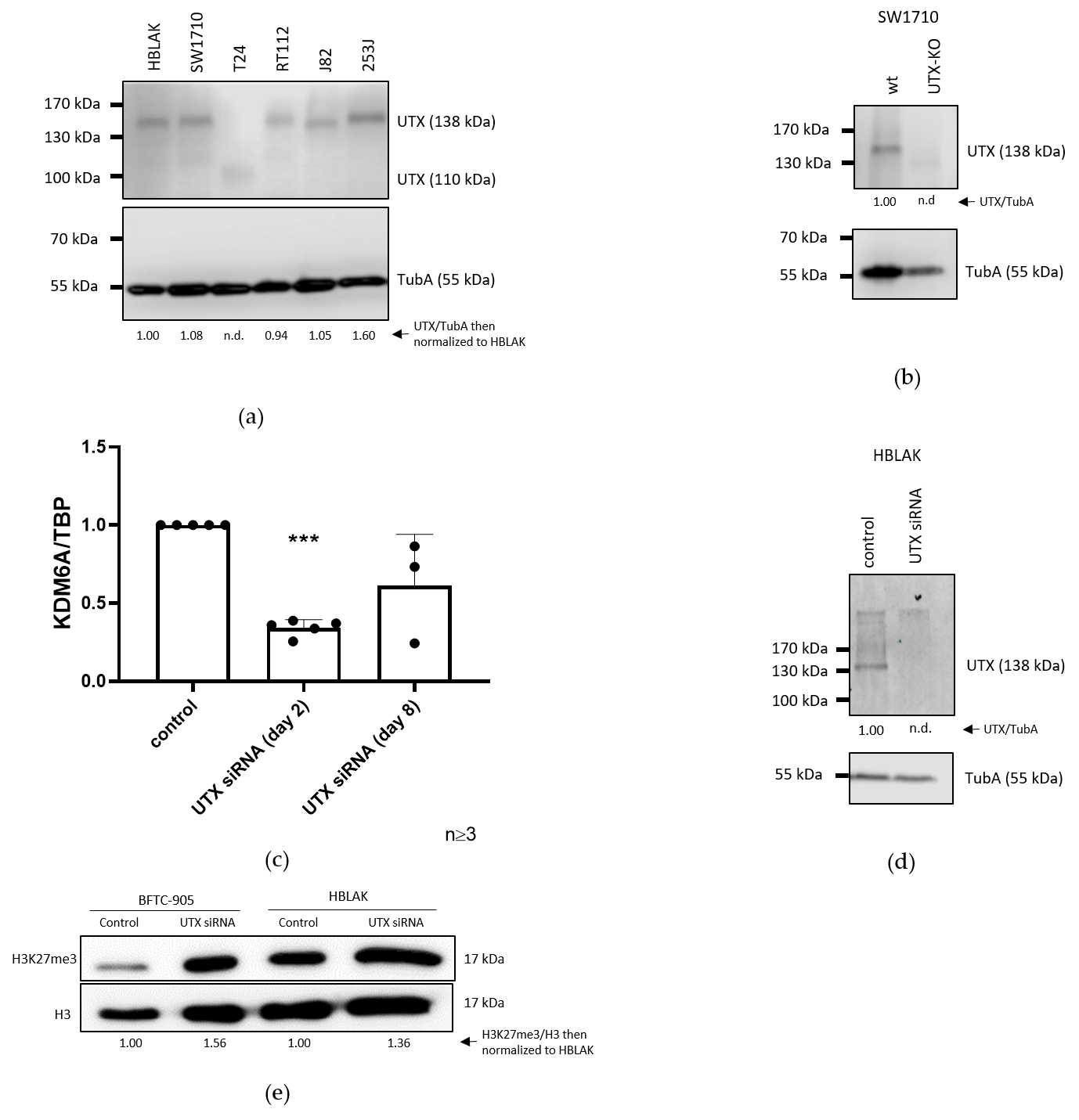

Supplement: Supplementary file 1 [file cancers-12-01023-s001.zip › Western Blots/Fig.S1n.tif]

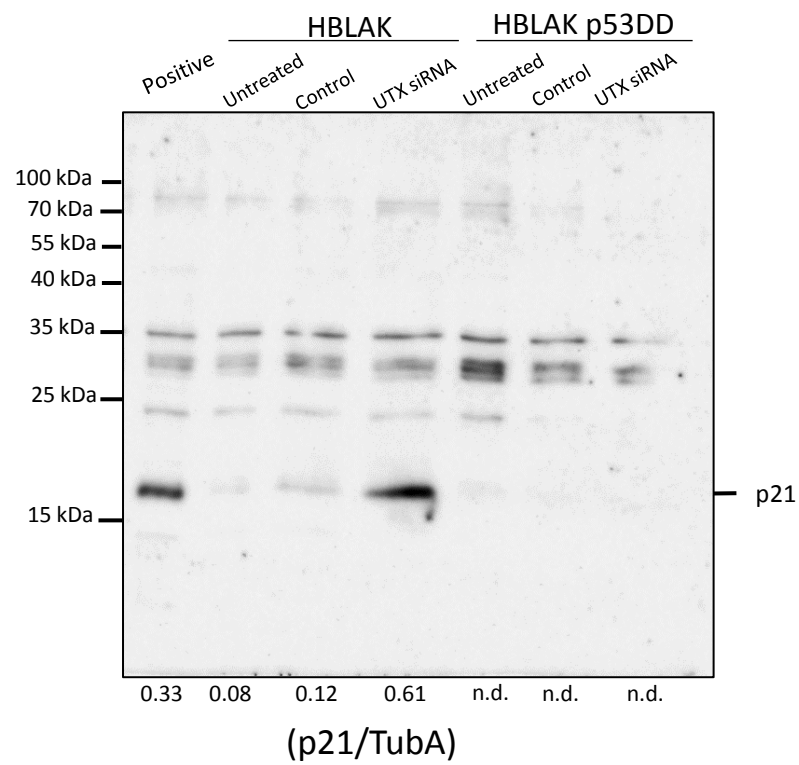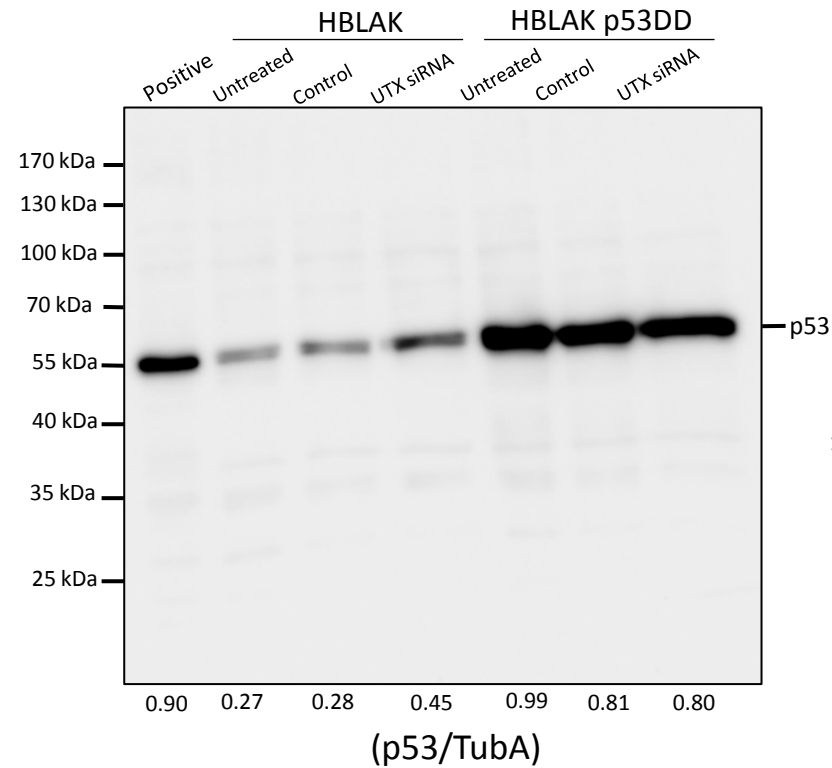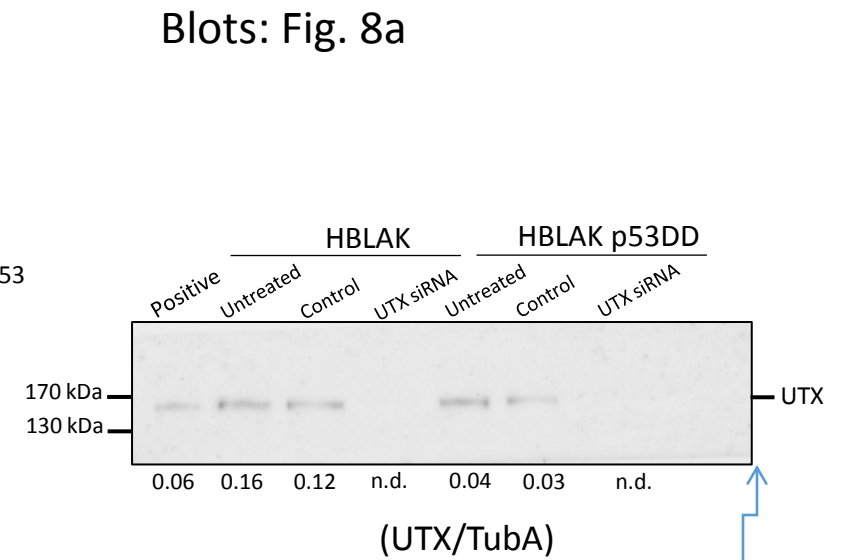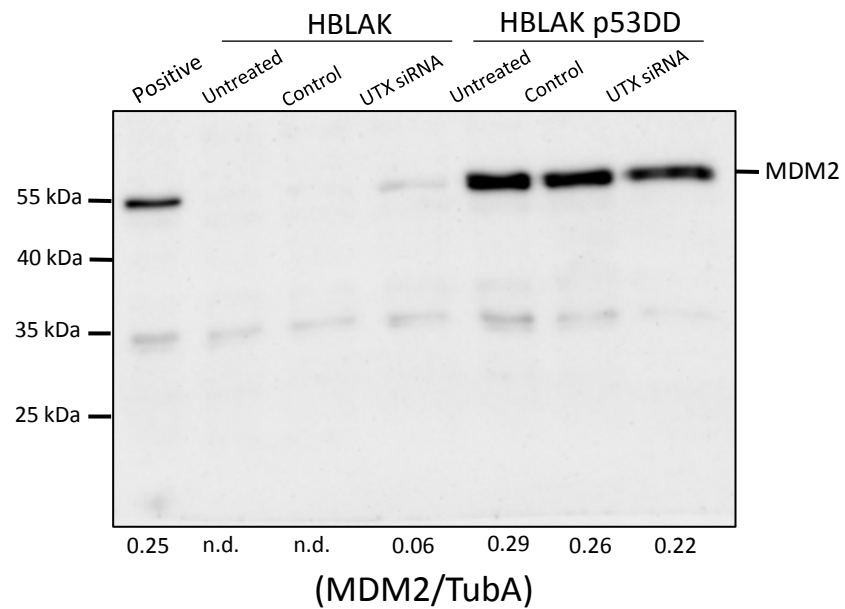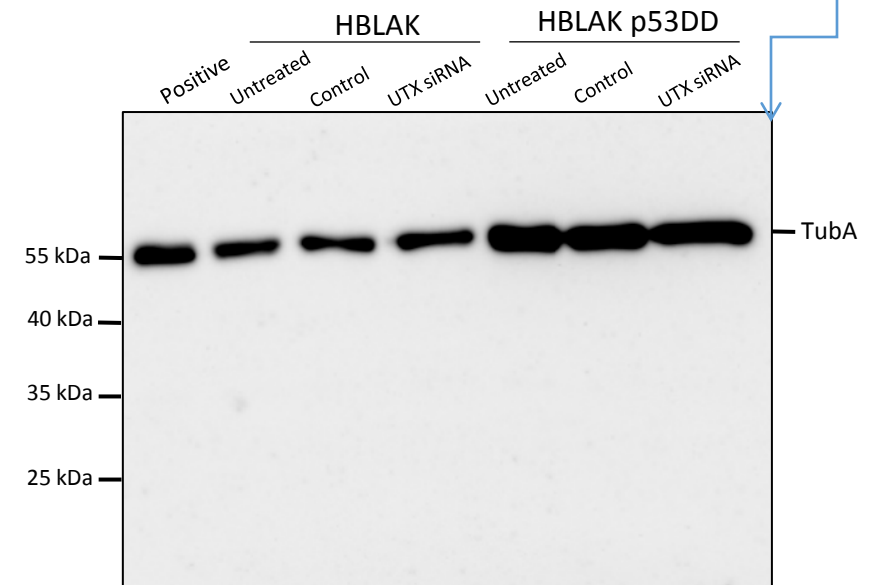

Cut

Blots: Fig. 9a

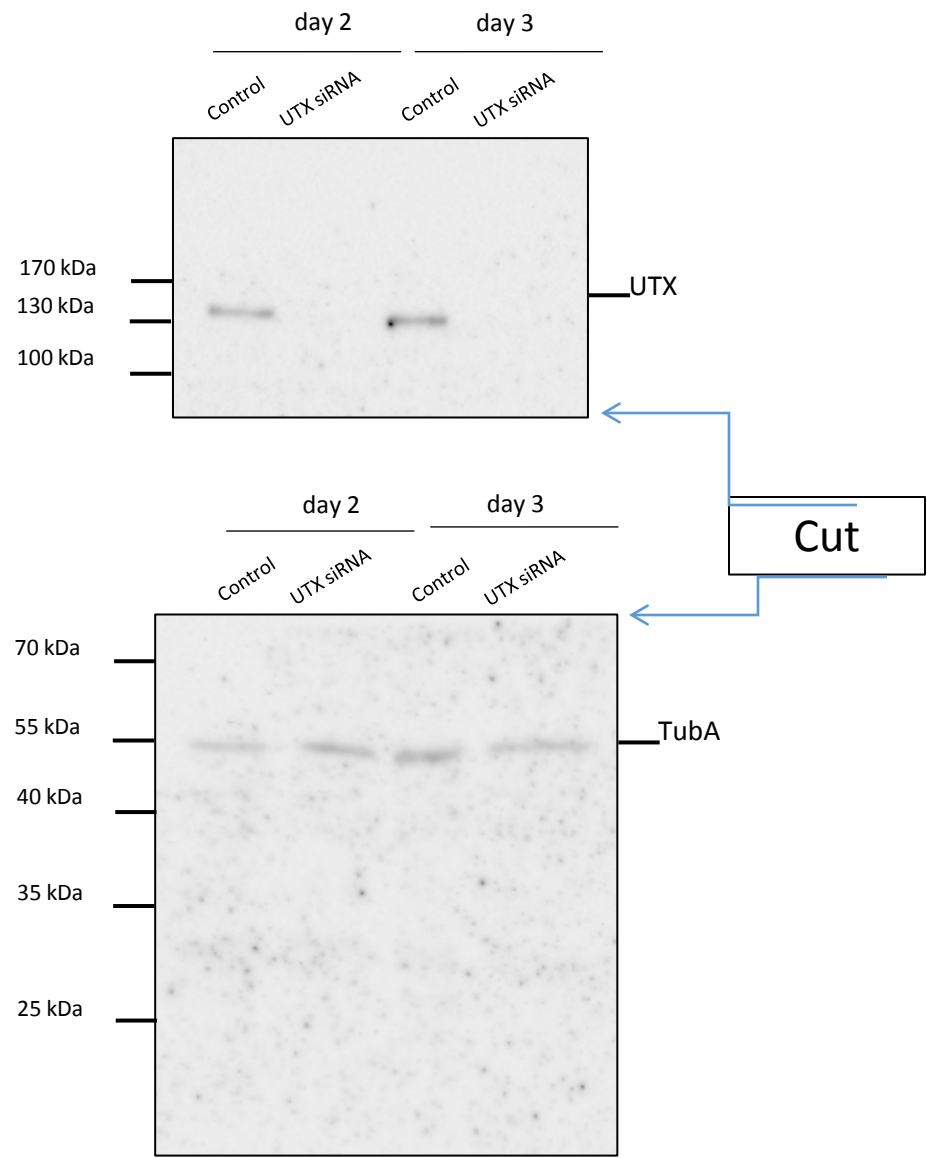

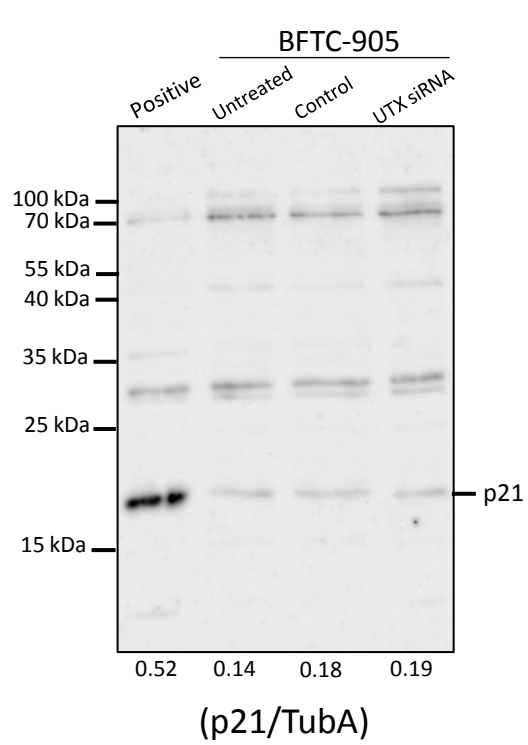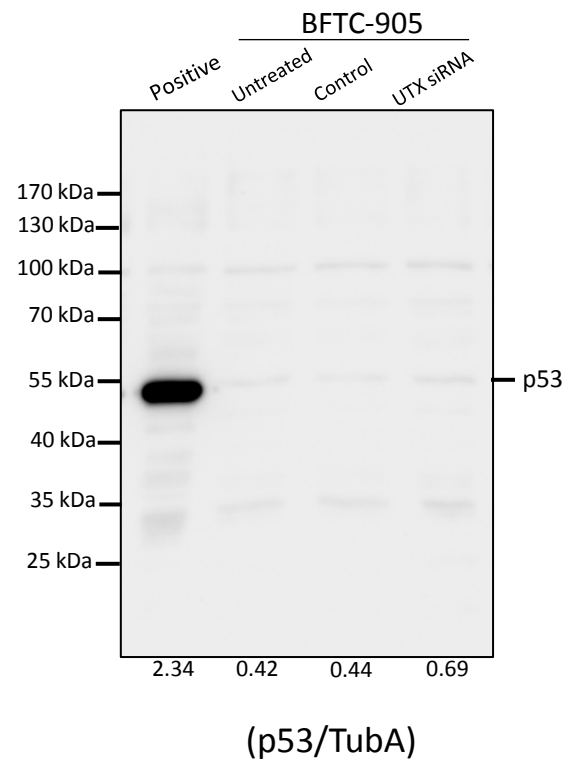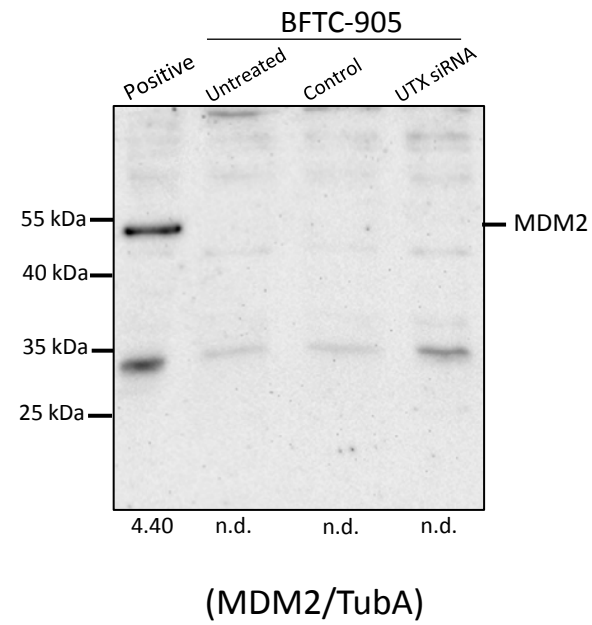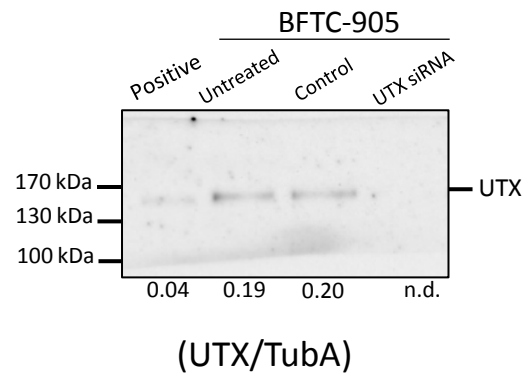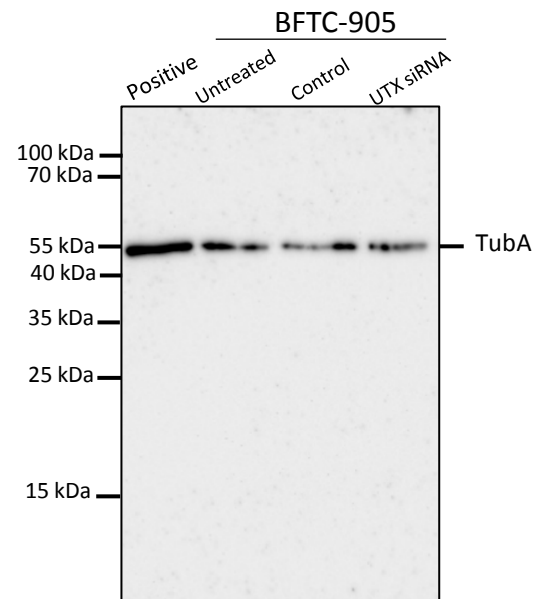

Blots: Fig. 9e

Blot: Fig. S1a

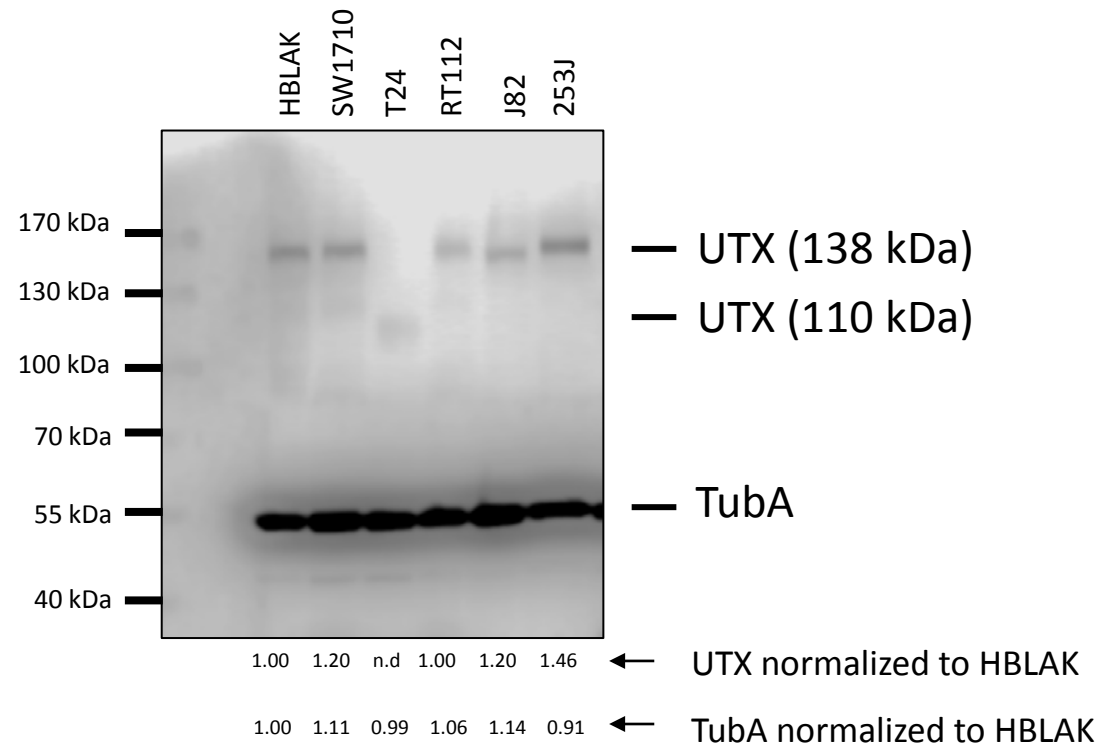

Blots: Fig. S1b

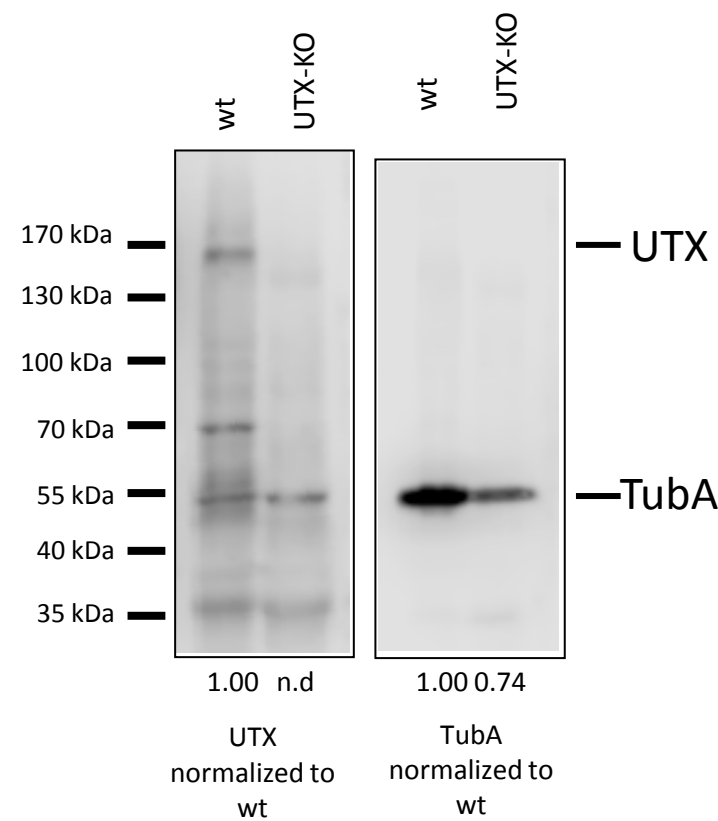

Blots: Fig. S1d

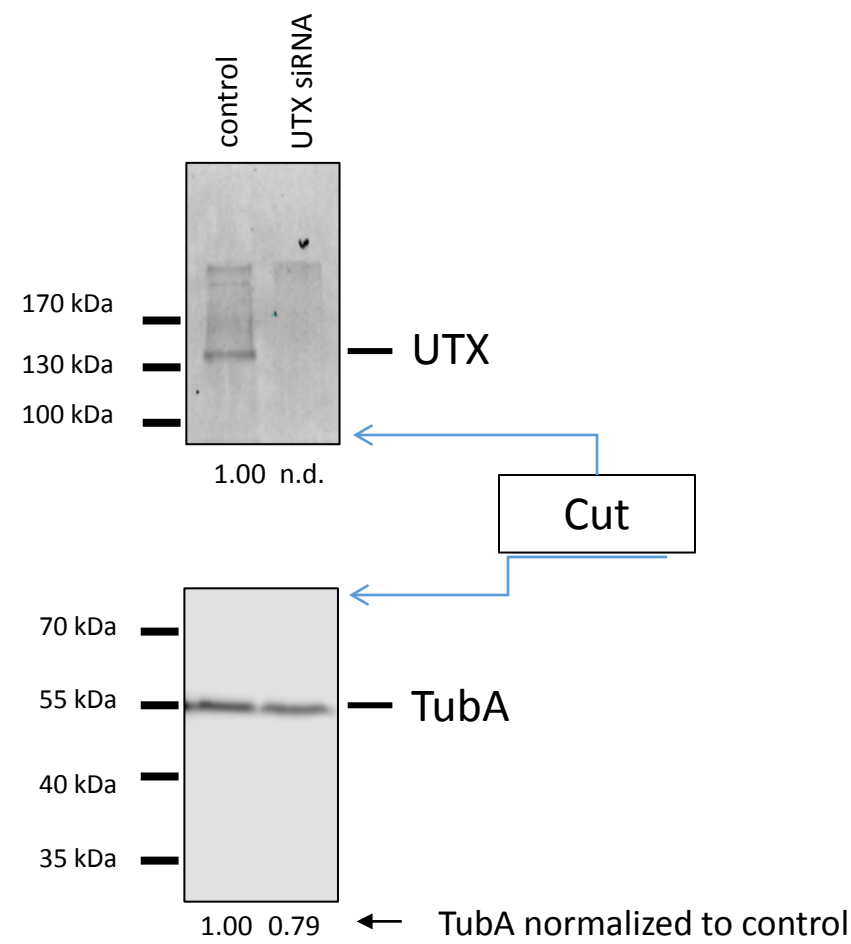

Blots: Fig. S1e

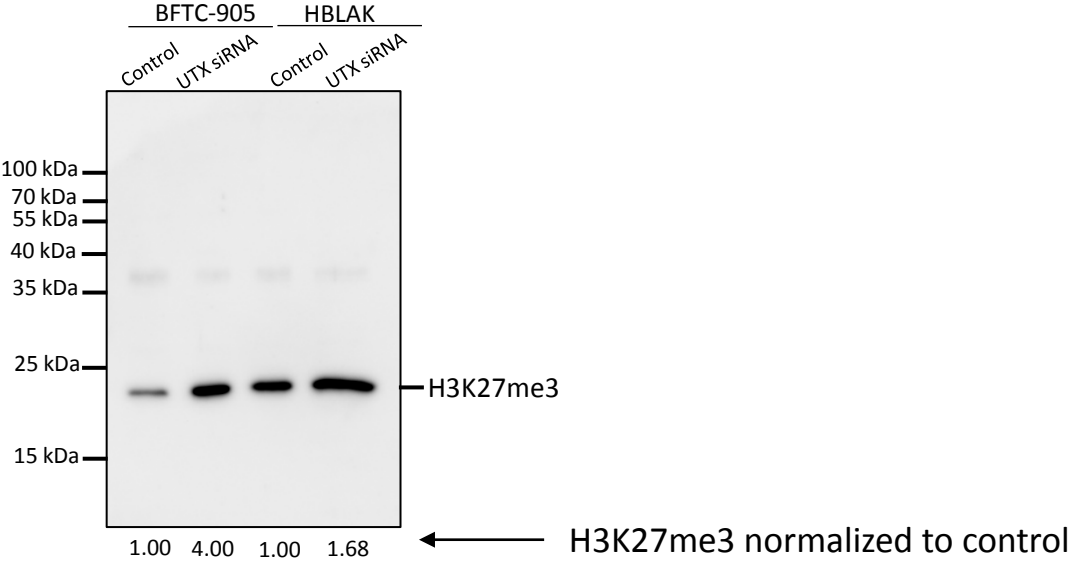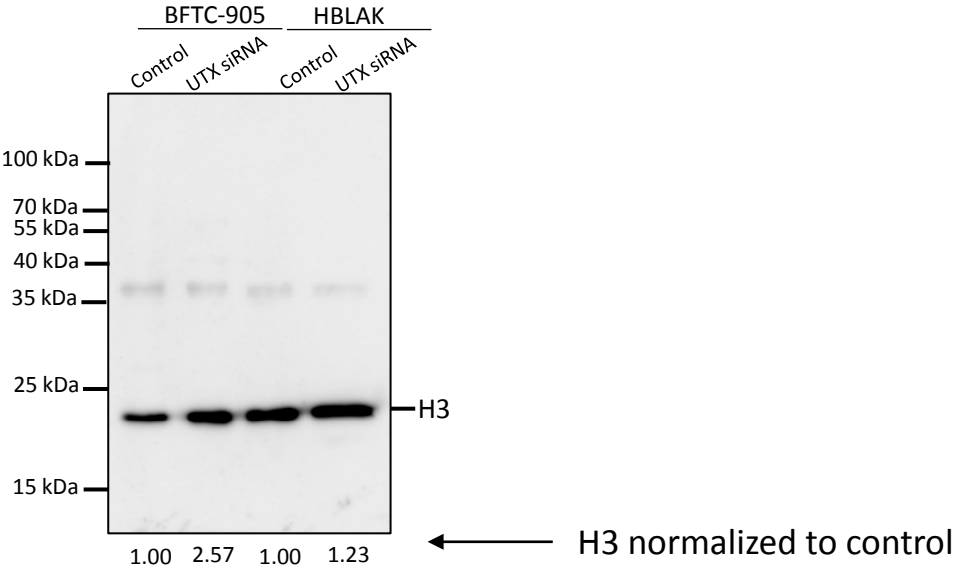

Supplement: Supplementary file 1 [file cancers-12-01023-s001.zip › Western Blots/original blots.pdf]

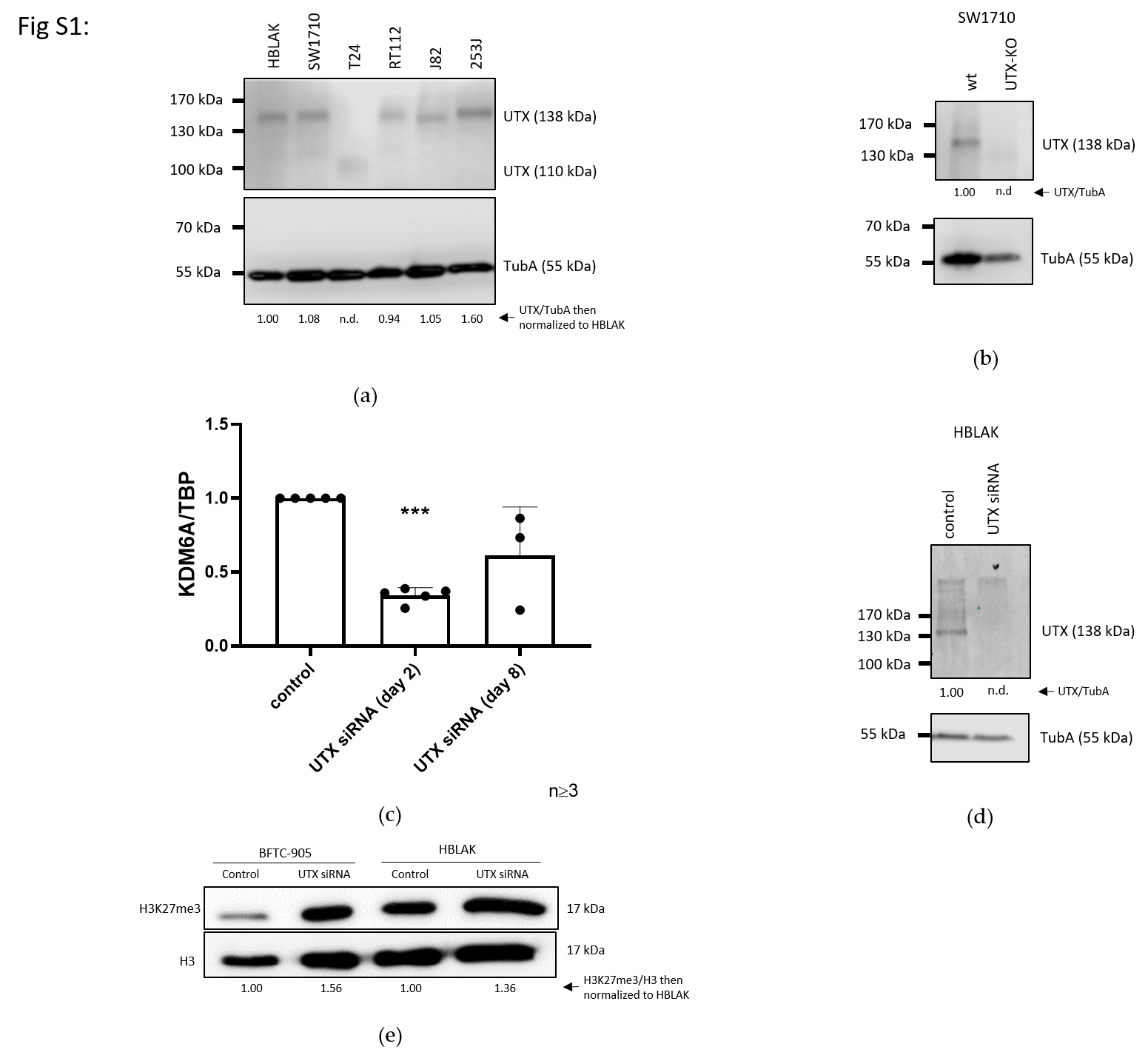

Supplement: Supplementary file 1 [file cancers-12-01023-s001.zip › Fig S1.tif]
